# Puzzling isotonic odd-even staggering of charge-radii in deformed rare earth nuclei

**DOI:** 10.1038/s41467-026-77471-7

**Published:** 2026-09-26

**Authors:** E. Takacs, H. Staiger, S. A. Blundell, N. Kimura, H. A. Sakaue, R. F. Garcia Ruiz, W. Nazarewicz, P.-G. Reinhard, C. A. Faiyaz, C. Suzuki, Dipti, I. Angeli, Y. Ralchenko, I. Murakami, D. Kato, Y. Nagai, R. Takaoka, Y. Miya, N. Nakamura

**Affiliations:** 1 Department of Physics and Astronomy, Clemson University, Clemson, SC USA; 2 National Institute for Fusion Science, Toki, Gifu Japan; 3 Univ. Grenoble Alpes, CEA, CNRS, Grenoble INP, IRIG, SyMMES, Grenoble, F-38000 France; 4 Massachusetts Institute of Technology, Cambridge, MA USA; 5 Harvard-MIT Center for Ultracold Atoms, Cambridge, MA USA; 6 Facility for Rare Isotope Beams and Department of Physics and Astronomy, Michigan State University, East Lansing, MI USA; 7 Institut für Theoretische Physik II, Universität Erlangen-Nürnberg, Erlangen, Germany; 8 Department of Experimental Physics, University of Debrecen, Debrecen, Hungary; 9 HUN-REN Institute for Nuclear Research, Debrecen, Hungary; 10 Department of Astronomy, University of Maryland, College Park, MD USA; 11 Astrophysics Science Division, NASA/GSFC, Greenbelt, MD USA; 12 Center for Research and Exploration in Space Science and Technology, NASA/GSFC, Greenbelt, MD USA; 13 Interdisciplinary Graduate School of Engineering Sciences, Kyushu University, Fukuoka, Japan; 14 Institute for Laser Science, The University of Electro-Communications, Tokyo, Japan

**Keywords:** Nuclear physics, Electronic structure of atoms and molecules

## Abstract

Isotonic (constant neutron number) systematics in the deformed rare-earth region have long suggested that nuclei with odd numbers of protons are more compact than their even-proton neighbors—except for Lu, whose recommended nuclear charge radius appeared anomalously large relative to Yb and Hf. We report a high-precision determination of the natural-abundance-averaged Lu-Yb charge-radius difference using extreme-ultraviolet spectroscopy of highly charged Na- and Mg-like ions, supported by high-accuracy relativistic atomic-structure calculations—a recently introduced method with the unique ability to measure inter-element charge-radius differences. Combined with muonic-atom and optical isotope-shift data, our results reestablish a pronounced odd-even staggering along the isotonic chain with neutron number ***N*** = **94**. Nuclear density functional theory calculations, including quantified uncertainties, fail to reproduce the large stagger, while successfully reproducing the charge-radii staggering along isotopic chains and in semi-magic nuclei. In this work, we resolve the Lu inversion anomaly and provide new constraints for experimental and theoretical studies of nuclear size.

## Abstract (web summary)

Enriching the sparse systematics across isotonic nuclear chains provides insight into the dependence of nuclear charge-radii with proton & neutron numbers. Here, the authors demonstrate high-precision measurements of the charge-radius difference between Lu and Yb with EUV spectroscopy of Na- & Mg-like ions, resolving the anomalous Lu charge radius and opening a path towards improved charge-radius systematics across isotonic chains.

## Introduction

The charge-radius is a fundamental nuclear observable that encodes the spatial distribution of nucleons within the nucleus, and offers insight into many properties of finite nuclei and nuclear matter^1–7^. In heavy, deformed nuclei, measurements of charge-radii provide key insights into how nuclear collectivity arises from the underlying shell structure^8–11^. These insights guide nuclear theory calculations, which are critical components of tests for beyond-the-Standard-Model physics using isotope shifts^12–14^ and have been used in muonic-atom charge-radius measurements to estimate nuclear polarization effects^15,16^.

Our current understanding of how nuclear size evolves with proton and neutron number relies primarily on comparisons across isotopic (constant proton number *Z*) chains that reveal striking variations in charge-radii^17^ and odd-even staggering in charge-radii (OESR) as a function of neutron number *N*^18^. In contrast, systematic comparisons across isotonic (constant *N*) chains remain sparse, because commonly used techniques, such as laser spectroscopy^6^, do not allow direct cross-element comparisons. This poses a major experimental obstacle to investigating the impact of isospin-breaking contributions and to constraining nuclear-matter properties using charge-radius measurements^19–23^. A more interconnected experimental picture would be valuable in extending modern nuclear models to the heavy, deformed rare-earth region^18,24–27^.

**Fig. 1:**
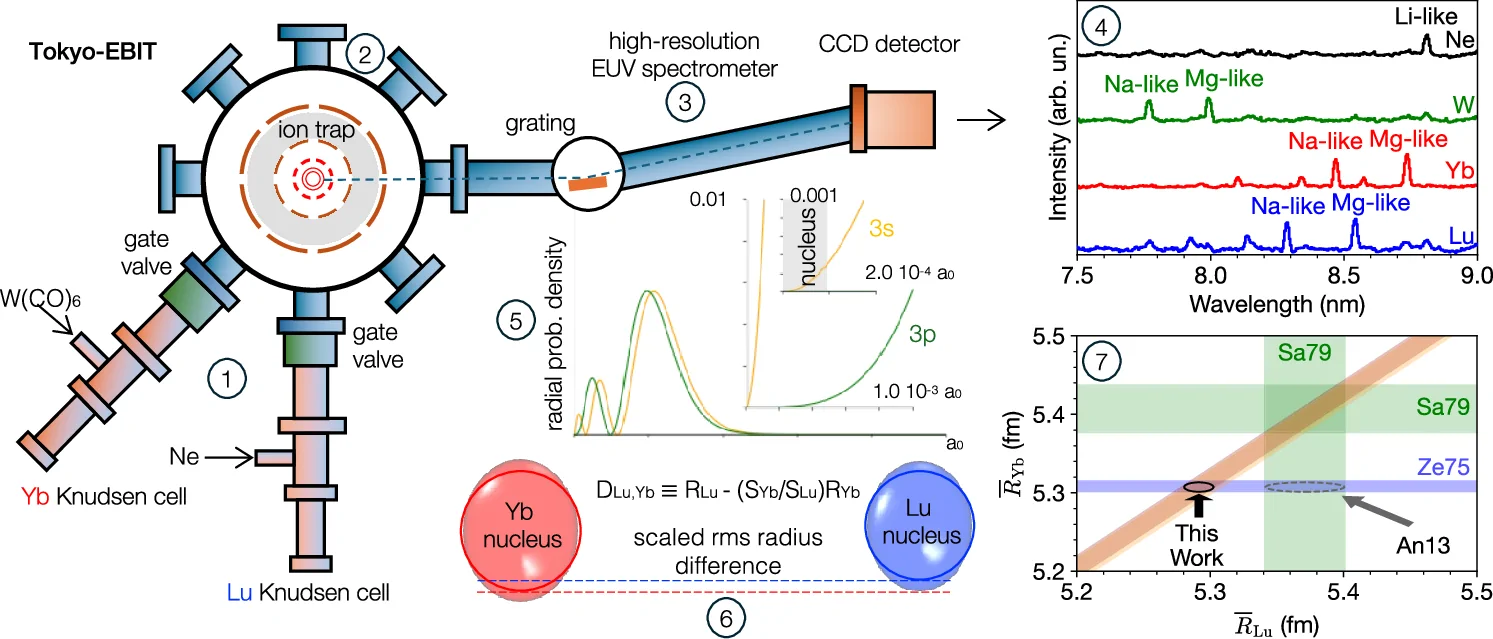
Overview of the experimental set-up and scaled charge radius difference extraction. **1** Schematic of the Tokyo-EBIT showing the ion trap, electron beam, and loading geometry for Lu and Yb atoms from independent Knudsen cells, with Ne and W(CO)_6_ injected for in-situ wavelength calibration; **2** the drift-tube region is immersed in a 3T magnetic field creating a 70*μm* by 2*cm* entrance-slit-like plasma for spectroscpy; **3** high-resolution grazing-incidence EUV spectrometer in which a variable-line-spacing grating disperses radiation onto a CCD detector; **4** summed EUV spectra from one day of data collection targeting the Na-like and Mg-like *D*_1_ line separations of Lu and Yb, calibrated using W *D*_1_ lines from Ref. ^56^ and Ne lines from the NIST ASD^64^; **5** illustrative hydrogenic 3*s* and 3*p* radial probability densities showing the sensitivity of the 3s electron to nuclear size, which enables extraction of (**6**) scaled rms-radius difference *D*_Lu,Yb_ ≡ *R*_Lu_ − (*S*_Yb_/*S*_Lu_)*R*_Yb_; (**7**) 1*σ* confidence regions for the naturally abundant Lu and Yb charge-radii, with electron-scattering (Sa79^31^) and muonic-atom (Ze75^29^) results shown as green and blue bands, the Angeli-Marinova 2013 evaluation^32^ shown by the dashed ellipse, and the highly charged ion constraints from this work shown in red (Na-like) and orange (Mg-like), together with the combined HCI+muonic-atom constraint indicated by the black ellipse.

In this work, we employ extreme ultraviolet (EUV) spectroscopy of highly charged Na-like ions^28^, combined with new measurements on Mg-like ions, to extract a high-precision difference in nuclear charge-radii between naturally abundant Lu and Yb elements. Figure 1 provides an overview of how these measurements yield nuclear charge-radius information: Lu and Yb neutral atoms are injected into an electron beam ion trap, where they are ionized to their Na-like and Mg-like charge states of interest. The emitted EUV line separations are recorded under identical conditions, with the spectrometer dispersion determined using well-known reference lines in this spectral region. The differing nuclear charge-radius sensitivities of the 3*s* and 3*p* electronic states allow these separations to be converted into a scaled Lu-Yb charge-radius difference through comparison with precise atomic-structure calculations. When anchored to muonic atom data for Yb^29^, our result provides a new value of the absolute nuclear charge-radii of Lu isotopes, including ^175^Lu. These absolute values may inherit the underlying work’s limitations, in this case those associated with charge-radius extraction from muonic-atom measurements in deformed nuclei (see Supplementary Note S1 for a discussion on previous absolute charge-radii measurements of Yb and Lu).

Our highly charged ion (HCI)-based scaled-difference technique provides a conceptually distinct approach to nuclear charge-radius determination in deformed nuclei. Due to the relatively low transition energies involved (≈100 eV vs. >1MeV), HCIs mostly avoid the interactions with excited nuclear states that complicate muonic-atom spectra^29,30^ and interpretation of electron scattering cross sections^31^ for deformed nuclei. Other systematics of radii from muonic atoms, such as nuclear model dependence, nuclear polarization corrections, and hyperfine splitting also have limited impacts on HCI radii information as shown in Supplementary Notes S3 and S4A. In this way, the present work establishes a cross-element, scaled-radius-difference method applicable to heavy, deformed nuclei. This technique provides a direct inter-element constraint on nuclear charge radii that enables systematic cross-checks of absolute determinations and can resolve inconsistencies between them.

Isotonic systematics in the deformed rare-earth region have long suggested that nuclei with an odd number of protons are more compact than their even-*Z* neighbors^32^. Lu has been a curious exception: its recommended charge-radius is anomalously larger than those of Yb and Hf, apparently contradicting the expected pattern of OESR^33^. With our threefold reduction in the uncertainty of the Lu radius, we now find that Lu is smaller than Yb, thereby restoring the OESR pattern in the *N* = 94 isotonic chain and resolving the decades-old Lu inversion anomaly.

The magnitude of this staggering in the *N* = 94 sequence is, moreover, unexpectedly large. While our nuclear Density Functional Theory (DFT) calculations correctly predict the small experimentally observed staggering in deformed isotopic and in heavy semi-magic isotopic and isotonic chains, they do not show the enhanced OESR suggested by the experimental data in the *N* = 94 chain.

To resolve this puzzle, precise measurements of the charge-radii of odd-*Z* deformed rare-earth nuclei are called for. The experimental method used in this work provides a promising avenue for such investigations.

## Results

### Atomic structure theory

The theoretical cornerstone of this work is the high precision attainable in atomic structure calculations for few-valence-electron ions, achieved through relativistic many-body perturbation theory (RMBPT) with quantum electrodynamic (QED) corrections^34^. Focusing on transition energy differences, rather than individual transition energies, significantly reduces both experimental and theoretical uncertainties, allowing for a precise determination of nuclear charge-radius differences (see Supplementary Notes S3B and S3C).

In addition to the Na-like calculation presented in Ref. ^28^, we have now achieved calculation of the significantly more complicated Mg-like transition energies. The strong state mixing within the valence shell corresponding to the principal quantum number *n* = 3 necessitates a hybrid RMBPT and configuration-interaction approach. In this method, an effective Hamiltonian—including the relevant QED contributions—is constructed within the *n* = 3 subspace and subsequently diagonalized. Further details of the method and the classification of RMBPT and QED contributions can be found in Methods, Supplementary Note S3, and Ref. ^34^.

Our central idea is to constrain the allowed set of nuclear-charge radii by requiring that the theoretical transition-energy difference, ${E}_{B}^{{{{\rm{th}}}}}({R}_{B})-{E}_{A}^{{{{\rm{th}}}}}({R}_{A})$, matches the experimentally measured difference, ${E}_{B}^{\exp }-{E}_{A}^{\exp }$, for two nuclides *A* and *B* with charge radii *R*_*A*_ and *R*_*B*_, respectively. Theoretical transition energies are well approximated by a linear expansion about an (in principle arbitrary) reference charge radius *R*_0_ chosen near the true radius: *E*^th^(*R*) = *E*^th^(*R*_0_) + *S*(*R* − *R*_0_), where $S\equiv {\left.\partial E/\partial R\right\vert }_{(R={R}_{0})}$ is the nuclear-size sensitivity coefficient^34^. Substituting this linearized form into ${E}_{B}^{{{{\rm{th}}}}}({R}_{B})-{E}_{A}^{{{{\rm{th}}}}}({R}_{A})={E}_{B}^{\exp }-{E}_{A}^{\exp }$ and rearranging then yields:

1 $$\begin{array}{l}{D}_{BA}\equiv {R}_{B}-\frac{{S}_{A}}{{S}_{B}}{R}_{A}=\left[{E}_{B}^{\exp }-{E}_{A}^{\exp }\right]/{S}_{B}-\\ -\left[{E}_{B}^{{{{\rm{th}}}}}({R}_{{B}_{0}})-{E}_{A}^{{{{\rm{th}}}}}({R}_{{A}_{0}})\right]/{S}_{B}+\left[{R}_{{B}_{0}}-\frac{{S}_{A}}{{S}_{B}}{R}_{{A}_{0}}\right],\end{array}$$

This equation shows that the key observable is not the simple radius difference *R*_*B*_ − *R*_*A*_, but rather a scaled (relative) radius difference, *D*_*BA*_ ≡ *R*_*B*_ − (*S*_*A*_/*S*_*B*_)*R*_*A*_. The ratio of sensitivity coefficients for A and B can be calculated accurately within atomic-structure theory (see Sec. S4 B). In Section S4, we generalize the derivation to include nuclear-model parameters, such as deformation, and we estimate second-order corrections to the underlying linear approximation, finding them to be negligible in practice.

The approximate linearity of transition energies with respect to changes in the nuclear radius implies that Eq. (1) also holds for two elements *A* and *B* if all nuclear parameters are replaced by their isotopic-abundance average. For example, ${R}_{A(B)}\to {\bar{R}}_{A(B)}\equiv {\sum }_{i}{w}_{iA(B)}{R}_{iA(B)}$ where *w*_*iA*(*B*)_ and *R*_*iA*(*B*)_ are the relative abundance^35^ and charge-radius^32^ of isotope *i* of element *A*(*B*). Details of the validity of this replacement, the treatment of higher-order nuclear moments, and the statistical combination of ${\bar{D}}_{BA}$ with absolute and relative charge-radii measurements are provided in Supplementary Note S4.

In earlier analyses^28,36^, Eq. (1) was solved for the charge-radius of a target isotope *B* using a reference isotope *A* as an anchor. The definition of the scaled radius difference removes dependence on a reference isotope and provides a clear representation of the experimental constraint these measurements provide (see Fig. 1(7)).

### Nuclear structure theory

The experimental results are compared with predictions from nuclear DFT^37^. We consider three energy density functionals: the Skyrme functional SV-min^38^ and two Fayans functionals, Fy(Δr, HFB)^39^ and Fy(IVP)^40^, which have nonstandard surface and pairing terms containing gradient couplings. Odd-*A* nuclei are computed with the blocking method^3^ by identifying one-quasiparticle configurations of odd nucleons through their quantum numbers: angular momentum projection on the symmetry axis and parity. In the calculations, we assumed the experimental assignments for the one-quasiparticle states in odd-*A* nuclei. The charge-radii were computed from deformed charge densities containing relativistic corrections (Darwin and spin-orbit term) and contributions from intrinsic nucleonic electric and magnetic form factors^27^. Further details are given in the Methods section.

### Experimental details

The measurements were carried out at the Tokyo Electron Beam Ion Trap (EBIT)^41,42^, operated at 10 keV electron beam energy and 100 mA beam current. Lu and Yb atoms were injected into the trap using a Knudsen cell source^43,44^, while Ne and W were co-injected to provide reference lines for wavelength calibration as depicted in Fig. 1(1). Extreme ultraviolet spectra of trapped ions were recorded with a flat-field grazing-incidence spectrometer^45^ equipped with a cooled CCD detector, achieving a resolution of 0.01 nm. Both Na-like and Mg-like charge states were studied, providing independent and complementary sensitivity to the nuclear charge-radius. A time-dependent wavelength calibration corrected for spectrometer drift, and all measurements of Lu, Yb, and calibration elements were interleaved to ensure identical EBIT conditions. Further details including ion injection and spectrometer operation are given in the Methods section.

### Restored OESR: resolving the Lu inversion anomaly

In this work, atoms of natural isotopic composition are injected into the electron beam ion trap. We determine the ${\bar{D}}_{{{{\rm{Lu}}}},{{{\rm{Yb}}}}}$ natural-abundance averaged scaled radius difference between Yb and Lu by combining experimentally measured Na-like and Mg-like energy differences with theoretical transition energy separations. The experimental differences are

2 $${E}_{{{{\rm{Lu}}}}}^{\exp,{{{\rm{Na}}}}}-{E}_{{{{\rm{Yb}}}}}^{\exp,{{{\rm{Na}}}}}=3.235(3)\,{{{\rm{eV}}}},$$

3 $${E}_{{{{\rm{Lu}}}}}^{\exp,{{{\rm{Mg}}}}}-{E}_{{{{\rm{Yb}}}}}^{\exp,{{{\rm{Mg}}}}}=3.229(3)\,{{{\rm{eV}}}},$$

while the corresponding theoretical separations, calculated using reference radii ${\bar{R}}_{{{{{\rm{Y\; b}}}}}_{0}}=5.3051\,{{{\rm{fm}}}}$ and ${\bar{R}}_{{{{{\rm{Lu}}}}}_{0}}=5.3701\,{{{\rm{fm}}}}$, obtained as natural-abundance averages from Ref. ^32^, are (see Table S1 in the SI)

4 $${E}_{{{{\rm{Lu}}}}}^{{{{\rm{th}}}},{{{\rm{Na}}}}}({\bar{R}}_{{{{{\rm{Lu}}}}}_{0}})-{E}_{{{{\rm{Yb}}}}}^{{{{\rm{th}}}},{{{\rm{Na}}}}}({\bar{R}}_{{{{{\rm{Y b}}}}}_{0}})=3.2144(6)\,{{{\rm{eV}}}},$$

5 $${E}_{{{{\rm{Lu}}}}}^{{{{\rm{th}}}},{{{\rm{Mg}}}}}({\bar{R}}_{{{{{\rm{Lu}}}}}_{0}})-{E}_{{{{\rm{Yb}}}}}^{{{{\rm{th}}}},{{{\rm{Mg}}}}}({\bar{R}}_{{{{{\rm{Y b}}}}}_{0}})=3.2103(7)\,{{{\rm{eV}}}}.$$

By comparing these experimental and theoretical values, we extracted the scaled difference in nuclear radii between Yb and Lu. With these results, the constraints we place on the scaled radius differences are:

6 $${\bar{D}}_{{{{\rm{Lu}}}},{{{\rm{Yb}}}}}^{{{{\rm{Na}}}}}=0.535(12)\,{{{\rm{fm}}}},\,\,{\bar{D}}_{{{{\rm{Lu}}}},{{{\rm{Yb}}}}}^{{{{\rm{Mg}}}}}=0.540(13)\,{{{\rm{fm}}}}.$$

For visualization purposes, these constraints are presented in Fig. 1(7), along with the muonic atom spectroscopy and elastic electron scattering results described in Supplementary Note S1. These measurements were converted to a natural-abundance averaged equivalent using the abundances of Ref. ^35^ and the optical isotope shift studies of Refs. ^46,47^.

To convert the natural-abundance-averaged values from Eq. (6) into information about individual charge-radii, we follow a generalized least-squares optimization procedure described in Section S4C of the SI that incorporates the Yb radii from the evaluation of Ref. ^32^, the isotopic differences from Refs. ^46,47^, and the natural abundances from Ref. ^35^. This yields a ^175^Lu radius of 5.291(6)(10) fm, where the first uncertainty reflects the reference radius and the second the present work, and an isotonic difference of ${R}_{{}^{175}{{{\rm{Lu}}}}}-{R}_{{}^{174}{{{\rm{Y\; b}}}}}=-0.017(10)\,{{{\rm{fm}}}}$. The conversion of natural-abundance averaged information into information about individual nuclides relies heavily on the high precision of optical isotope shift data that, relative to our precision, effectively fix the isotopic charge radius differences. It is this rigidity of the Yb and Lu chains that allows a difference in the natural-abundance average of the chains to be translated into a difference between isotopes.

## Discussion

The extracted isotonic difference supports the existence of normal OESR along the Yb–Lu–Hf triplet, in direct contrast to the inverted OESR seen in recommended values^32,48^. This isotonic decrease in charge-radius is consistent with previous electron scattering work^31^ that disagreed with the muonic atom results for Yb^29^. One resolution to the tension displayed in Fig. 1(7) is that both charge-radii from Sa79 are overestimated due to some common systematic effect.

**Fig. 2:**
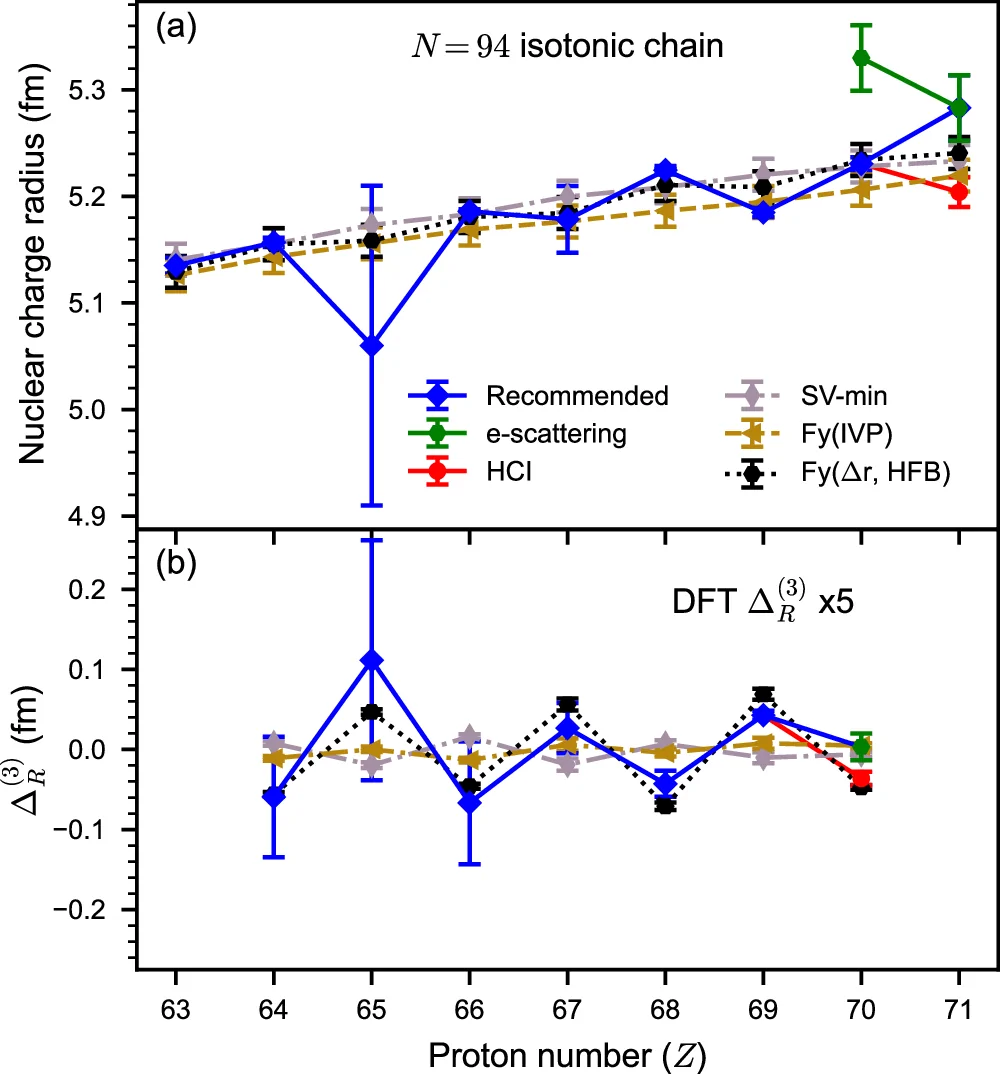
Improved charge radius and odd-even staggering from highly charged ions. **a** charge-radii along the *N* = 94 isotonic chain. The recommended values, anchored mostly by muonic atom spectroscopy, are taken from Ref. ^32^, with the exception of ${R}_{{}^{160}{{{\rm{Dy}}}}}$, which was taken from Ref. ^48^ due to an apparent overestimation of uncertainty in Ref. ^32^. Also shown is the measurement from this work (solid red circles) alongside electron-scattering data^31^ for *Z* = 70–71 (green hexagons). Nuclear DFT predictions using SV-min, Fy(IVP), and Fy(Δ*r*,HFB) functionals are also shown. All error bars represent 1*σ* uncertainties. **b** The corresponding OESR (Eq. (7)). The calculated values of OESR and their uncertainties are multiplied by five for the sake of visibility.

For the sake of comparison with nuclear DFT calculations, we extracted the experimental nuclear charge-radii for the long *N* = 94 isotonic chain from Ref. ^32^ and included ${R}_{{}^{165}{{{\rm{Lu}}}}}=5.204(14)\,{{{\rm{fm}}}}$ inferred from our work and optical isotope shift measurements^46^, see Fig. 2(a). The systematic behavior of charge radii across the *N* = 94 to *N* = 104 isotonic chains, including the influence of our updated ^175^Lu radius, is shown in Supplementary Fig. 7. In the *N* = 94 calculation, we assumed the experimental assignments for the one-quasiproton states in odd-*Z* nuclei using the Nilsson labels^49^: [413]5/2^+^ for ^157^Eu, [411]3/2^+^ for ^159^Tb, [523]7/2^−^ for ^161^Ho, [411]1/2^+^ for ^163^Tm, and [404]7/2^+^ for ^165^Lu. This sequence of one-quasiproton band-heads is reproduced by theory^8^, except for the inverted ordering of the close-lying [523]7/2^−^ and [411]1/2^+^ levels at *Z* = 67 and *Z* = 69.

To quantify the OESR, Fig. 2(b) shows the three-point charge-radii difference for the *N* = 94 chain:

7 $${\Delta }_{R}^{(3)}=\frac{1}{2}\left({R}_{N+1}-2{R}_{N}+{R}_{N-1}\right).$$

Theoretically, the OESR is primarily influenced by nucleonic pairing and quadrupole polarization effects; see, e.g., Refs. ^10,18,26,50–52^. In particular, the pairing effect is the main source of the OESR in spherical semi-magic nuclei. Strong deformation effects in OESR have been seen in transitional nuclei that are deformation-soft, in which an odd nucleon in a deformation-driven orbital can significantly polarize the nuclear shape^52^. For well-deformed nuclei, such as those investigated in this work, such shape-polarization effects are considerably weaker^49^.

**Fig. 3:**
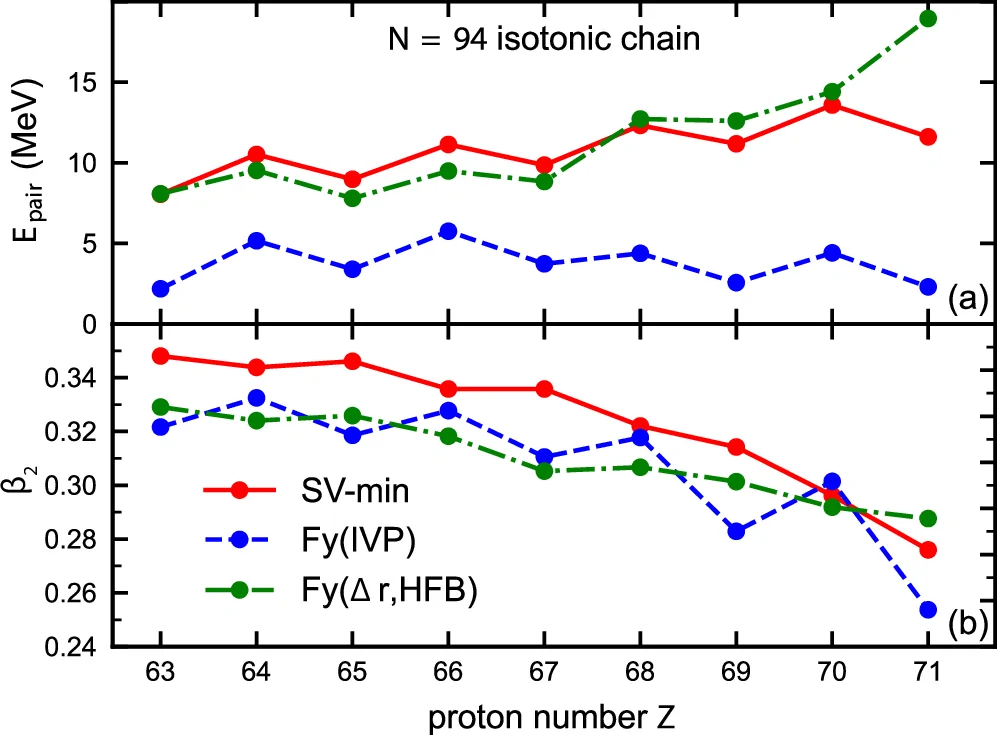
Predicted pairing energy and quadrupole deformation for *N* = 94 isotones. **a** The pairing energy *E*_pair_, which is not an observable, shows significant model dependence, while all the EDF parametrizations used reproduce very well the observed OES of binding energy. Along the isotonic *N* = 94 chain, the staggering in *E*_pair_ is weak; it reflects the usual reduction of pairing correlations in odd-*A* nuclei due to blocking. **b** The quadrupole deformation *β*_2_ is deduced from the calculated proton quadrupole moment and can be related to experimental quadrupole moments; see Ref. ^8^ for examples. The OES of quadrupole deformations is also weak: small fluctuations of *β* seen in odd-*Z* reflect different shape-polarization effects of different one-quasiproton orbits ^8,49^ and reduced pairing. Since pairing is a symmetry-restoring correlation^65,66^, larger values of *E*_pair_ are expected to reduce *β*_2_, and vice versa.

In general, the absolute charge-radii recommended in Ref. ^32^ are fairly well reproduced by our DFT models, considering experimental and theoretical uncertainties. However, all models predict a much smaller OESR than the OESR shown by the recommended charge-radii combined with our HCI constraint. We found the odd-even staggering in the calculated pairing energy and quadrupole deformation *β*_2_ to be weak along the *N* = 94 sequence (see Fig. 3), which is consistent with the weaker predicted OESR.

**Fig. 4:**
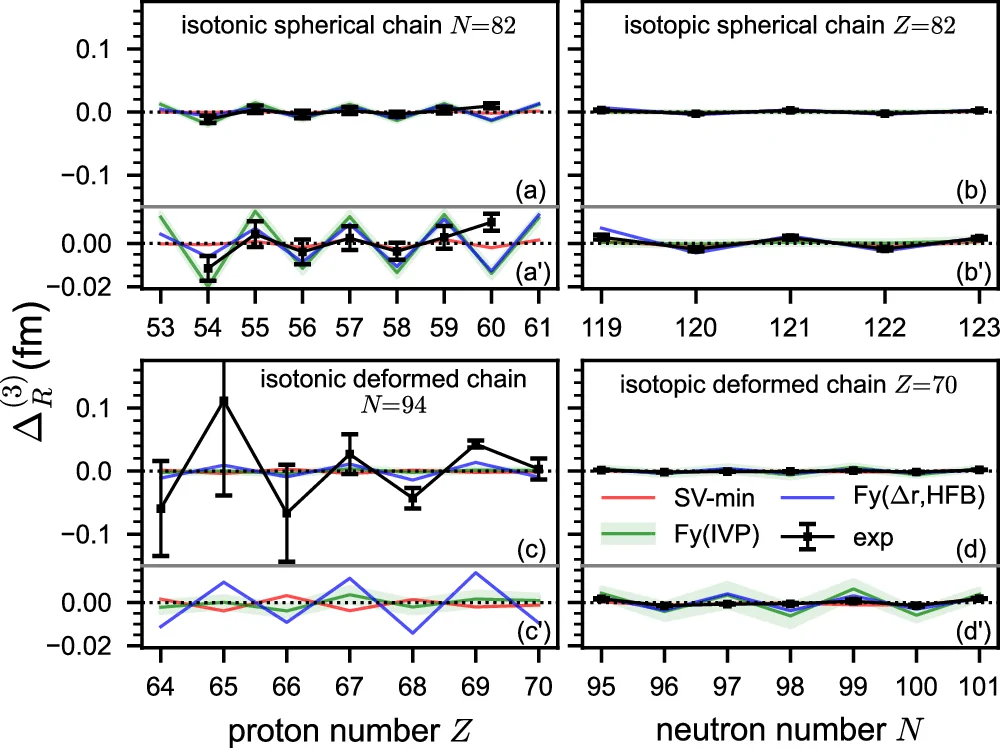
Comparison of experimental OESR along isotonic and isotopic chains. Experimental recommended values^32^ and DFT predictions for ${\Delta }_{R}^{(3)}$ in isotonic (**a**, **c**) and isotopic (**b**, **d**) chains of selected spherical (top) and deformed (bottom) nuclei. Note the dramatic change of the experimental OESR magnitude for the deformed *N* = 94 chain when compared to other cases. The details of OESR are shown in panels (a',b',c',d') and Supplementary Fig. 6. The shaded region of Fy(IVP) represents the sum of statistical and systematic uncertainties. Experimental error bars are the 1*σ* uncertainty from Ref. ^32^.

To get a better insight into the discrepancy seen in Fig. 2b, Fig. 4 shows experimental and theoretical values of ${\Delta }_{R}^{(3)}$ for isotonic *N* = 82 and 92 and isotopic *Z* = 82 and 70 chains. The semi-magic nuclei with *Z* = 82 or *N* = 82 are spherical, while the rare-earth chains are strongly deformed. The DFT calculations reproduce OESR for spherical sequences, as well as for the deformed Yb isotopic chain, with the model dependence of the predicted magnitude of OESR being primarily attributed to different pairing functionals employed. For the *N* = 94 nuclei, the experimental OESR is very large: it exceeds the experimental OESR values in other sequences displayed by a factor of ≈30. To see whether the large OESR is a local property of the *N* = 94 chain, Supplementary Fig. 5a shows ${\Delta }_{R}^{(3)}$ for *N* = 94−104 even-*N* isotonic sequences and confirms that the situation shown in Fig. 4c is in fact typical for the whole region. For better depiction of theoretical trends and uncertainties, see Supplementary Fig. 6.

Our measurement of the ^175^Lu − ^174^Yb scaled nuclear charge-radius difference provides a unique constraint on the charge-radius surface and resolves a longstanding anomaly in the rare-earth region. The radius constraints from the Na-like and Mg-like *D*_1_ line energy differences agree with each other, supporting the internal consistency of the analysis and increasing the overall credibility of the measurement and theoretical framework.

The observed OESR along the *N* = 94 isotonic chain is substantially more pronounced than predicted by current state-of-the-art nuclear density functional theory, even though these approaches perform well for isotopic chains and in other regions of the nuclear chart. This discrepancy highlights a potentially important deficiency in present theoretical descriptions of deformed rare-earth nuclei. Alternatively, aspects of the muonic-atom (and electron scattering) analysis in this highly deformed region may merit re-examination, including contributions associated with nuclear-model dependence and nuclear polarization.

Improving the precision of the Tb and Ho charge-radii, or equivalently their radius difference relative to Dy (*Z* = 66), would provide useful insight into the pronounced experimental isotonic OESR seen in Figs. 2 and 4c, and Supplementary Fig. 5a. Given the complexity of muonic-atom spectra in the highly deformed region 60≤*Z*≤77 ^48^, highly charged ion spectroscopy could provide a particularly powerful approach for establishing radius trends in this region.

## Methods

### Electron beam ion trap

The measurements reported in this work were performed using the Tokyo-EBIT at the University of Electro-Communications in Tokyo^41,42^. The EBIT consists of three main components: an electron gun, a cryogenic trap region with drift tubes, and an electron collector. During the present experiment, the EBIT was operated at an electron beam energy of 10 keV and an electron beam current of 100 mA. The electron beam was compressed by a magnetic field of 3T, generated by a superconducting split coil, which focused the electrons into a narrow radius to achieve a high current density for efficient ionization.

The compressed, high-density electron beam ionizes elements injected through a side port, and the resulting ions are confined in the trapping region, which consists of a series of cylindrical drift tube electrodes. Ion trapping is achieved by the combination of a potential well created by the applied drift tube voltages and the space-charge potential of the compressed electron beam. The trapped ions are further ionized through successive electron impact. For the present measurements, a potential well of 300V was used.

To alternately introduce Lu and Yb atoms, two Knudsen cells^43,44^ were mounted on the side ports of the Tokyo EBIT. Each cell was aligned with a side window of the drift tube assembly, and the atomic beam was collimated through a pair of 1 mm pinholes to ensure ballistic injection into the trap region. This method avoids the complexity of pulsed injection and is particularly effective for metallic elements with moderate vapor pressures. The operating temperatures were 1000 °C for Lu and 350 °C for Yb.

A stainless steel pipe with a stop valve was attached to the side of the Knudsen cell for ytterbium injection. This pipe was used to introduce W(CO)_6_ and Ne gases for observing the spectra of highly charged W and Ne ions for wavelength calibration^53–56^. Ne was also introduced during the Lu and W measurements as a source of light, low-charge state ions to evaporatively cool the injected heavy elements. This cooling effect enhanced the population of Na-like and Mg-like charge states, thereby increasing the signal rate and improving the quality of the spectroscopic data. To observe Ne spectra, the trapped ions were dumped at a frequency of 5 Hz to prevent the accumulation of heavier elements, whereas no dumping was applied during the observations of Lu, Yb, and W.

### EUV Spectroscopy

Extreme ultraviolet spectra of the trapped ions were recorded using a flat-field grazing incidence spectrometer^45^. The spectrometer employs a variable-line-spacing grating optimized for the 5–20 nm wavelength range and is equipped with a Peltier-cooled CCD detector for high-resolution, low-noise imaging. The system is capable of operating in both first- and second-order diffraction modes, enabling flexible trade-offs between spectral coverage and resolution.

The spectrometer is mounted on one of the radial observation ports of the Tokyo EBIT, with a direct line of sight to the trap center without any additional filters used to record spectra. The electron beam itself serves as the effective entrance slit, and the spectrometer’s geometry ensures that the spatial extent of the ion cloud does not degrade the spectral resolution. The spectral resolution achieved in the present measurements was approximately 0.01 nm, consistent with the instrumental width reported in previous EBIT studies^57^. This resolution was sufficient to resolve the Na-like and Mg-like *D*_1_ (3*p*^2^*P*_1/2_ → 3*s*^2^*S*_1/2_ and 3*s*3*p*^3^*P*_1_ → 3*s*^2 1^*S*_0_) transitions of interest.

### Measurement sequence

The data acquisition campaign was conducted over the course of five days, structured around the operational constraints of the Tokyo EBIT’s cryogenic system. The EBIT required liquid helium refills approximately every seven hours, with two fills scheduled per day. This dictated the available measurement windows and segmented the experiment into two approximately 7-hour sessions per day.

Within each session, spectra were recorded in 10-minute intervals for different injected elements. The sequence was executed in the following order: 30 minutes of ytterbium, 70 minutes of lutetium, 50 minutes of tungsten, and 50 minutes of neon. This sequence maintained roughly equal statistics in the Yb and Lu lines of interest. All of the element’s injected were of natural abundance^35^ due to the challenge of obtaining large enough isotopically pure samples. The Ne spectra were acquired with a 5 Hz ion dump rate to reduce contaminant W species and to provide frequent in situ wavelength calibration references. This alternating sequence ensured that each element was measured under comparable EBIT conditions, including electron beam energy, current, and magnetic field.

### Data analysis

Experimentally, the analysis of the extreme ultraviolet spectra focused on extracting the shift in Na-like 3*p*^2^*P*_1/2_ → 3*s*^2^*S*_1/2_ and Mg-like 3*s*3*p*^3^*P*_1_ → 3*s*^2 1^*S*_0_ *D*_1_ transition energies from our measured spectra. The data recorded were processed using a combination of image correction, spectral fitting, and wavelength calibration techniques.

Raw CCD images included a slight tilt of the spectral lines, occasional cosmic ray hits, and background signal from stray light. Line tilt was corrected by rotating the images, while cosmic ray artifacts were removed using contour detection algorithms, ensuring clean spectra for subsequent analysis. For the background, we implemented a flat-field correction technique based on a sliding minimum filter.

Spectral lines were fit using a Gaussian profile superimposed on a constant background, typically within a narrow window of ±8 pixels around the peak. Calibration was performed using well-known Ne and W emission lines. A time-dependent calibration model was implemented, which allowed for a linear drift in time over the course of each day, but with a shared spatial dispersion for all days. To assist in determining the time drift, a term in the calibration loss function was added that penalized spread in the calculated wavelengths of the Na- and Mg-like transitions of Lu and Yb.

The Lu-Yb *D*_1_ transition energy shifts in each injection session were extracted for both Na-like and Mg-like charge states. Both the calibration of the spectrometer and the line statistics contribute to the experimental shift uncertainties. To account for any unresolved systematic uncertainties, all experimental uncertainties were multiplied by the square root of our calibration ${\chi }_{\nu }^{2}$^58^. These experimentally determined energy differences were then compared to theoretical predictions from our relativistic many-body perturbation theory calculations^28,34^. See Section S2 of the SI for further details.

### RMBPT+QED atomic structure calculations

*D*_1_ transition energies in Na-like ions^34^ are calculated using relativistic many-body perturbation theory (RMBPT)^59^ combined with an ab initio treatment of QED^60^. The RMBPT terms include the Coulomb and Breit interaction up to third order, based on a zeroth-order Breit-Coulomb Dirac-Fock (BC-DF) potential for the 10-electron Ne-like core. The QED sector includes the one-loop self-energy and vacuum polarization computed in the local direct part of the BC-DF potential; to these are added approximate leading-order correlation corrections, including core-relaxation and valence-core exchange QED terms. Two-loop QED corrections are added phenomenologically.

For Mg-like ions, we construct an effective Hamiltonian for the strongly interacting *n* = 3 valence-shell complex up to third order in RMBPT, and add approximate one-loop QED terms computed in a similar way to those for Na-like ions. This approach generalizes one previously applied to the *n* = 4 complex of Zn-like ions^61^. We also include new valence-valence QED terms, not present for Na-like ions.

Further details, numerical results, and a discussion of theoretical uncertainties can be found in Section S3 of the SI.

### Nuclear density functional theory calculations

All three density functional parameterizations used have been calibrated to the same large set of nuclear observables from Ref. ^38^, including binding energies, charge-radii, diffraction radii and surface thickness, odd-even binding energy differences, and spin-orbit splitting. The Fy(Δr, HFB) calibration dataset additionally incorporates selected differential charge-radius data from the Ca isotopic chain. The dataset of Fy(IVP) also contains selected charge-radii differences along the semimagic Sn and Pb isotopic chains, and employs a more general isospin-dependent pairing term. The deformed nuclei are computed with the axial DFT solver SkyAx^62^. The uncertainties of the calibrated model parameters are propagated to calculated observables, such as charge-radii, by means of the linear regression technique^38,63^.

## Supplementary information

Supplementary Information

Transparent Peer Review file

## Data Availability

The data supporting the findings of this study are contained in the Article and Supplementary Information. The underlying numerical data are available from the corresponding authors upon request. Raw CCD images and calibration data are not deposited in a public repository due to their large data volume and are available from the corresponding authors upon request.

## Code availability

These research codes and associated scripts are not maintained as a documented or user-ready software package. However, the analysis procedures, including the generalized least-squares approach described in Supplementary Note S4C, can be made available from the corresponding authors upon request.

## References

[1] Drell, S. D. Nuclear radius and nuclear forces. *Phys. Rev.* **100**, 97–112 10.1103/PhysRev.100.97 (1955).

[2] Bohr, A. & Mottelson, B. R.Nuclear Structure, vol. I (W. A. Benjamin, New York, 1969).

[3] Ring, P., Schuck, P. The Nuclear Many-Body Problem. Springer-Verlag: Berlin, 1980.

[4] Ekström, A. et al. Accurate nuclear radii and binding energies from a chiral interaction. *Phys. Rev. C.* **91**, 051301 10.1103/PhysRevC.91.051301 (2015).

[5] Reinhard, P.-G. & Nazarewicz, W. Nuclear charge and neutron radii and nuclear matter: Trend analysis in Skyrme density-functional-theory approach. *Phys. Rev. C.* **93**, 051303 10.1103/PhysRevC.93.051303 (2016).

[6] Nörtershäuser, W. & Moore, I. D. Nuclear Charge Radii, 1-70 (Springer, Singapore, 2023).

[7] Miyagi, T. Nuclear radii from first principles. *Front. Phys.* **13**, 1581854 10.3389/fphy.2025.1581854 (2025).

[8] Reinhard, P.-G. & Nazarewicz, W. Statistical correlations of nuclear quadrupole deformations and charge Radii. *Phys. Rev. C.* **106**, 014303 10.1103/PhysRevC.106.014303 (2022).

[9] Zerguine, S., Van Isacker, P. & Bouldjedri, A. Consistent description of nuclear charge radii and electric monopole transitions. *Phys. Rev. C.* **85**, 034331 10.1103/PhysRevC.85.034331 (2012). 18764177

[10] Geldhof, S. et al. Impact of nuclear deformation and pairing on the charge radii of palladium isotopes. *Phys. Rev. Lett.* **128**, 152501 10.1103/PhysRevLett.128.152501 (2022). 35499902

[11] Warbinek, J. et al. Smooth trends in fermium charge radii and the impact of shell effects. *Nature* **634**, 1075–1079 10.1038/s41586-024-08062-z (2024). PMC11525170 39478208

[12] Hur, J. et al. Evidence of two-source King plot nonlinearity in spectroscopic search for new boson. *Phys. Rev. Lett.* **128**, 163201 10.1103/PhysRevLett.128.163201 (2022). 35522508

[13] Berengut, J. C. & Delaunay, C. Precision isotope-shift spectroscopy for new physics searches and nuclear insights. *Nat. Rev. Phys.* **7**, 119–125 10.1038/s42254-024-00793-2 (2025).

[14] Door, M. et al. Probing new bosons and nuclear structure with ytterbium isotope shifts. *Phys. Rev. Lett.* **134**, 063002 10.1103/PhysRevLett.134.063002 (2025). 40021164

[15] Sun, Z. et al. ^208^Pb nuclear charge radius revisited: Closing the fine-structure-anomaly gap. *Phys. Rev. Lett.* **135**, 163002 10.1103/h3xz-xdxr (2025). 41172215

[16] Beyer, K. A. et al. Modern approach to muonic X-Ray spectroscopy demonstrated through the measurement of stable Cl radii 10.48550/arXiv.2506.08804 (2025).

[17] Garcia Ruiz, R. F. et al. Unexpectedly large charge radii of neutron-rich calcium isotopes. *Nat. Phys.* **12**, 594–598 10.1038/nphys3645 (2016).

[18] de Groote, R. P. et al. Measurement and microscopic description of odd-even staggering of charge radii of exotic copper isotopes. *Nat. Phys.* **16**, 620–624 10.1038/s41567-020-0868-y (2020).

[19] Pineda, S. V. et al. Charge radius of neutron-deficient ^54^Ni and symmetry energy constraints using the difference in mirror pair charge radii. *Phys. Rev. Lett.* **127**, 182503 10.1103/PhysRevLett.127.182503 (2021). 34767412

[20] Reinhard, P.-G. & Nazarewicz, W. Information content of the differences in the charge radii of mirror nuclei. *Phys. Rev. C.* **105**, L021301 10.1103/PhysRevC.105.L021301 (2022).

[21] Bano, P., Pattnaik, S. P., Centelles, M., Viñas, X. & Routray, T. R. Correlations between charge radii differences of mirror nuclei and stellar observables. *Phys. Rev. C.* **108**, 015802 10.1103/PhysRevC.108.015802 (2023).

[22] Novario, S. J., Lonardoni, D., Gandolfi, S. & Hagen, G. Trends of neutron skins and radii of mirror nuclei from first principles. *Phys. Rev. Lett.* **130**, 032501 10.1103/PhysRevLett.130.032501 (2023). 36763401

[23] Ohayon, B. Critical evaluation of reference charge radii and applications in mirror nuclei. *Data Nucl. Data Tables* **165**, 101732 10.1016/j.adt.2025.101732 (2025).

[24] Yang, D., Rong, Y.-T., An, R. & Shi, R.-X. Potential signature of new magicity from universal aspects of nuclear charge radii. *Phys. Rev. C.* **110**, 064314 10.1103/PhysRevC.110.064314 (2024).

[25] An, H., Zhang, Z. & Wang, B. A novel approach to nuclear charge radii using machine learning. *Nucl. Phys. A* **1004**, 122026 10.1016/j.nuclphysa.2020.122026 (2020).

[26] Reinhard, P.-G. & Nazarewicz, W. Toward a global description of nuclear charge radii: Exploring the Fayans energy density functional. *Phys. Rev. C.* **95**, 064328 10.1103/PhysRevC.95.064328 (2017).

[27] Reinhard, P.-G. & Nazarewicz, W. Nuclear charge densities in spherical and deformed nuclei: Toward precise calculations of charge radii. *Phys. Rev. C.* **103**, 054310 10.1103/PhysRevC.103.054310 (2021).

[28] Hosier, A. et al. Determination of nuclear charge radius by extreme-ultraviolet spectroscopy of Na-like ions. *Phys. Rev. Res.* **7**, L012024 10.1103/PhysRevResearch.7.L012024 (2025).

[29] Zehnder, A. et al. Charge parameters, isotope shifts, quadrupole moments, and nuclear excitation in muonic 170-174,176-Yb. *Nucl. Phys. A* **254**, 315–340 10.1016/0375-9474(75)90219-5 (1975).

[30] Fricke, G. et al. Nuclear ground state charge radii from electromagnetic interactions.* Data Nucl. Data Tables* **60**, 177–285 10.1006/adnd.1995.1007 (1995).

[31] Sasanuma, T. Electron Scattering from Deformed Heavy Nuclei. Ph.D. thesis, Massachusetts Institute of Technology (1979).

[32] Angeli, I. & Marinova, K. P. Table of experimental nuclear ground state charge radii: An update.* Data Nucl. Data Tables* **99**, 69–95 10.1016/j.adt.2011.12.006 (2013).

[33] Angeli, I. et al. N and Z dependence of nuclear charge radii. *J. Phys. G: Nucl. Part. Phys.* **36**, 085102 10.1088/0954-3899/36/8/085102 (2009).

[34] Gillaspy, J. D., Osin, D., Ralchenko, Y., Reader, J. & Blundell, S. A. Transition energies of the D lines in Na-like ions. *Phys. Rev. A* **87**, 062503 10.1103/PhysRevA.87.062503 (2013).

[35] Berglund, M. & Wieser, M. E. Isotopic compositions of the elements 2009 (IUPAC Technical Report). *Pure Appl. Chem.* **83**, 397–410 10.1351/PAC-REP-10-06-02 (2011).

[36] Hosier, A. et al. Absolute nuclear charge radius by Na-like spectral line separation in high-Z elements. *J. Phys. B: Mol. Opt. Phys.* **57**, 195001 10.1088/1361-6455/ad717b (2024).

[37] Bender, M., Heenen, P.-H. & Reinhard, P.-G. Self-consistent mean-field models for nuclear structure. *Rev. Mod. Phys.* **75**, 121–180 10.1103/RevModPhys.75.121 (2003).

[38] Klüpfel, P., Reinhard, P.-G., Bürvenich, T. J. & Maruhn, J. A. Variations on a theme by Skyrme: A systematic study of adjustments of model parameters. *Phys. Rev. C.* **79**, 034310 10.1103/PhysRevC.79.034310 (2009).

[39] Miller, A. J. et al. Proton superfluidity and charge radii in proton-rich calcium isotopes. *Nat. Phys.* **15**, 432–436 10.1038/s41567-019-0416-9 (2019).

[40] Karthein, J. et al. Electromagnetic properties of indium isotopes illuminate the doubly magic character of 100Sn. *Nat. Phys.* **20**, 1719–1725 10.1038/s41567-024-02612-y (2024).

[41] Currell, F. J. et al. A new versatile electron-beam ion trap. *J. Phys. Soc. Jpn.* **65**, 3186–3192 10.1143/JPSJ.65.3186 (1996).

[42] Nakamura, N. et al. An overview of the Tokyo electron beam ion trap. *Phys. Scr.* **1997**, 362–364 10.1088/0031-8949/1997/T73/119 (1997).

[43] Yamada, C. et al. Injection of metallic elements into an electron-beam ion trap using a Knudsen cell. *Rev. Sci. Instrum.* **77**, 066110 10.1063/1.2216867 (2006).

[44] Nakamura, N. et al. Injection of various metallic elements into an electron beam ion trap: Techniques needed for systematic investigations of isoelectronic sequences. *Rev. Sci. Instrum.* **71**, 684–686 10.1063/1.1150260 (2000).

[45] Ohashi, H., Yatsurugi, J., Sakaue, H. A. & Nakamura, N. High-resolution extreme ultraviolet spectrometer for an electron beam ion trap. *Rev. Sci. Instrum.* **82**, 083103 10.1063/1.3618686 (2011). 21895229

[46] Georg, U. et al. Laser spectroscopy investigation of the nuclear moments and radii of lutetium isotopes. *Eur. Phys. J. A* **3**, 225–235 10.1007/s100500050172 (1998).

[47] Kawasaki, A., Kobayashi, T., Nishiyama, A., Tanabe, T. & Yasuda, M. Isotope-shift analysis with the 4*f*^14^6*s*^2^^1^*S*_0_ – 4*f*^13^5*d*6*s*^2^(*J* = 2) transition in ytterbium. *Phys. Rev. A* **109**, 062806 10.1103/PhysRevA.109.062806 (2024).

[48] Fricke, G. & Heilig, K.Nuclear Charge Radii, vol. 20 of Landolt-Bornstein - Group I Elementary Particles, Nuclei and Atoms (Springer Materials).

[49] Nazarewicz, W., Riley, M. & Garrett, J. Equilibrium deformations and excitation energies of single-quasiproton band heads of rare-earth nuclei. *Nucl. Phys. A* **512**, 61–96 10.1016/0375-9474(90)90004-6 (1990).

[50] Koszorús, Á et al. Charge radii of exotic potassium isotopes challenge nuclear theory and the magic character of N=32. *Nat. Phys.* **17**, 439–443 10.1038/s41567-020-01136-5 (2021).

[51] Le Blanc, F. et al. Large odd-even radius staggering in the very light platinum isotopes from laser spectroscopy. *Phys. Rev. C.* **60**, 054310 10.1103/PhysRevC.60.054310 (1999).

[52] Marsh, B. A. et al. Characterization of the shape-staggering effect in mercury nuclei. *Nat. Phys.* **14**, 1163–1167 10.1038/s41567-018-0292-8 (2018).

[53] Kramida, A. & Buchet-Poulizac, M.-C. Energy levels and spectral lines of Ne VII. *Eur. Phys. J. D.* **38**, 265–276 10.1140/epjd/e2006-00025-3 (2006).

[54] Kramida, A. & Buchet-Poulizac, M.-C. Energy levels and spectral lines of Ne VIII. *Eur. Phys. J. D.* **39**, 173–188 10.1140/epjd/e2006-00122-3 (2006).

[55] Ralchenko, Y. et al. EUV spectra of highly-charged ions W^54+^ − W^63+^ relevant to ITER diagnostics. *J. Phys. B: Mol. Opt. Phys.* **41**, 021003 10.1088/0953-4075/41/2/021003 (2008).

[56] Gillaspy, J. D. et al. Measurement of the D-line doublet in high-Z highly charged sodiumlike ions. *Phys. Rev. A* **80**, 010501(R) 10.1103/PhysRevA.80.010501 (2009).

[57] Koike, F. et al. Z-dependent crossing of excited-state energy levels in highly charged gallium-like lanthanide atomic ions. *Phys. Rev. A* **105**, 032802 10.1103/PhysRevA.105.032802 (2022).

[58] Birge, R. T. The calculation of errors by the method of least squares. *Phys. Rev.* **40**, 207–227 10.1103/PhysRev.40.207 (1932).

[59] Johnson, W. R., Blundell, S. A. & Sapirstein, J. Many-body perturbation-theory calculations of energy levels along the sodium isoelectronic sequence. *Phys. Rev. A* **38**, 2699–2706 10.1103/PhysRevA.38.2699 (1988). 9900685

[60] Blundell, S. A. Calculations of the screened self-energy and vacuum polarization in Li-like, Na-like, and Cu-like ions. *Phys. Rev. A* **47**, 1790–1803 10.1103/PhysRevA.47.1790 (1993). 9909131

[61] Blundell, S. A. Calculation of QED corrections in highly charged Zn-like ions. *Can. J. Phys.* **87**, 55–65 10.1139/p08-065 (2009).

[62] Reinhard, P.-G., Schuetrumpf, B. & Maruhn, J. The axial Hartree-Fock + BCS code SkyAx. *Comput. Phys. Commun.* **258**, 107603 (2021).

[63] Dobaczewski, J., Nazarewicz, W. & Reinhard, P.-G. Error estimates of theoretical models: a guide. *J. Phys. G* **41**, 074001 10.1088/0954-3899/41/7/074001 (2014).

[64] Kramida, A., Ralchenko, Y., Reader, J. & NIST ASD Team. NIST Atomic Spectra Database (ver. 5.12), [Online]. Available: https://physics.nist.gov/asd [2026, July 8]. National Institute of Standards and Technology, Gaithersburg, MD (2024).

[65] Reinhard, P.-G. & Otten, E. W. Transition to deformed shapes as a nuclear Jahn-Teller effect. *Nucl. Phys. A* **420**, 173 10.1016/0375-9474(84)90437-8 (1984).

[66] Nazarewicz, W. Microscopic origin of nuclear deformations. *Nucl. Phys. A* **574**, 27 – 49 10.1016/0375-9474(94)90037-X (1994).

