# Supplementary material for: Puzzling isotonic odd-even staggering of charge-radii in deformed rare earth nuclei
Source: Nat Commun. 2026 Sep 26;17:11159. doi: 10.1038/s41467-026-77471-7 (PMC13710950; doi:10.1038/s41467-026-77471-7)
Supplement: Supplementary file 1 [file 41467_2026_77471_MOESM1_ESM.pdf]

Supplementary Information for: Puzzling Isotonic Odd-Even Staggering of Charge Radii in Deformed Rare Earth Nuclei

H. Staiger,[1] E. Takacs,[1,2] S. A. Blundell,[3] N. Kimura,[2] H. A. Sakaue,[2] R. F. Garcia Ruiz,[4,5] W. Nazarewicz,[6] P.-G. Reinhard,[7] C. A. Faiyaz,[1] C. Suzuki,[2] Dipti,[1] I. Angeli,[8,9] Yu. Ralchenko,[10,11,12] I. Murakami,[2] D. Kato,[2,13] Y. Nagai,[14] R. Takaoka,[14] Y. Miya,[14] and N. Nakamura[14]

[1]*Department of Physics and Astronomy, Clemson University, Clemson, SC 29634, USA*[*]
[2]*National Institute for Fusion Science, Toki, Gifu 509-5292, Japan*
[3]*Univ. Grenoble Alpes, CEA, CNRS, Grenoble INP, IRIG, SyMMES, 38000 Grenoble, France*
[4]*Massachusetts Institute of Technology, Cambridge, Massachusetts 02139, USA*
[5]*Harvard-MIT Center for Ultracold Atoms, Cambridge, Massachusetts 02138, USA*
[6]*Facility for Rare Isotope Beams and Department of Physics and Astronomy, Michigan State University, East Lansing, Michigan 48824, USA*
[7]*Institut für Theoretische Physik II, Universität Erlangen-Nürnberg, Staudtstrasse 7, D-91058 Erlangen, Germany*
[8]*Department of Experimental Physics, University of Debrecen, Hungary, 4026*
[9]*HUN-REN Institute for Nuclear Research, ATOMKI, Bem ter 18/c, H-4026 Debrecen, Hungary*
[10]*Department of Astronomy, University of Maryland, College Park, MD 20742, USA*
[11]*Astrophysics Science Division, NASA/GSFC, Greenbelt, MD 20771, USA*
[12]*Center for Research and Exploration in Space Science and Technology, NASA/GSFC, Greenbelt, MD 20771, USA*
[13]*Interdisciplinary Graduate School of Engineering Sciences, Kyushu University, Fukuoka 816-8580, Japan*
[14]*Institute for Laser Science, The University of Electro-Communications, Tokyo 182-8585, Japan*

[*] Contacts: etakacs@clemson.edu, hstaige@clemson.edu, kimura.naoki@nifs.ac.jp, n_nakamu@ils.uec.ac.jp

CONTENTS

Supplementary Notes 3

S1. Previous Absolute Charge Radii Measurements 3

S2. Data Analysis 3
- A. Spectral Processing and Corrections 4
- B. Line Fitting and Calibration 4
- C. Transition Energy Shift Extraction 6

S3. Atomic-Structure Theory 7
- A. Transition energies of Na- and Mg-like ions 7
- B. Uncertainty in theoretical transition energies 9
- C. Suppression of uncertainty in transition-energy differences 11

S4. Nuclear Charge Radii Determination 12
- A. Scaled Charge Radius Difference 12
- B. Sensitivity to Nuclear Density Parameters 14
- C. Charge Radii Optimization Procedure 16
- D. Results 18

Supplementary Figures 21

Supplementary References 24

Supplementary Notes

S1. PREVIOUS ABSOLUTE CHARGE RADII MEASUREMENTS

Absolute radius data are scarce for the lutetium isotopes. Aside from Sasanuma's 1979 Ph.D. thesis [S1], which reported a radius of 5.37(3) fm for ^{175}Lu from electron scattering measurements assuming spherical nuclear charge density, the only other known measurement is from Suzuki's 1968 Ph.D. thesis [S2], which employed muonic atom spectroscopy. That study reported Fermi distribution parameters ($c = 6.25(10)$ fm, $t = 2.07(30)$ fm) but did not provide a direct value for the nuclear charge radius. Although Suzuki's result was included in early muonic atom compilations such as that of Engfer *et al.* [S3], it saw limited use in later charge radius evaluations due to the adoption of a spherical charge distribution for the deformed Lu nucleus. Consequently, Sasanuma's thesis result remained the primary reference point for radii in the Lu isotopic chain [S4, S5].

The most precise values for the stable Yb isotopes come from a muonic atom spectroscopy experiment by Zehnder *et al.* in 1975 [S6]. Although the original publication did not report uncertainties for the root-mean-square (*rms*) radii, later evaluations estimated uncertainties of 0.006 fm [S7] and 0.002 fm [S5]. Additional muonic atom studies include the Ph.D. theses of Adler (1975) [S8] and Bernhardt (1992) [S9]. These studies reported similar radii to each other and Zehnder's work, with the primary uncertainties stemming from uncertainties in the nuclear polarization calculations.

Several elastic electron scattering studies were also conducted using Yb. The earliest was by Cooper *et al.* in 1976 [S10], which measured ^{176}Yb, followed by Creswell's 1977 Ph.D. thesis [S11] on the same isotope. While Cooper *et al.* unfortunately did not extract a charge radius, Creswell reported a charge radius of 5.443 fm. These works employed a deformed Fermi distribution rather than a model-independent parameterization such as the Fourier-Bessel expansion [S12]. In contrast, Sasanuma's 1979 Ph.D. thesis measured the radii of both ^{174}Yb and ^{175}Lu to nominal uncertainties of 0.03 fm using a Fourier–Bessel expansion [S1]. Sasanuma's radius of 5.41(3) fm is significantly higher than the 5.308(6) fm reported by Zehnder *et al.* from muonic atom spectroscopy for ^{174}Yb [S6].

As a result, recent evaluations of nuclear charge radii have relied primarily on the muonic atom measurement by Zehnder *et al.* to anchor the Yb isotopes, and on Sasanuma's electron scattering thesis for the Lu isotopes [S4, S5]. This combination led to an apparent inversion of the expected odd-even staggering across the isotonic Yb–Lu–Hf triplets: the charge radius of the odd-Z Lu isotope appeared significantly larger than those of its even-Z neighbors—opposite to the systematics observed throughout the rare earth region [S4, S5]. Our new determination of the Yb–Lu radius difference reduces its uncertainty substantially, allows for a new determination of the Lu radius, and resolves a discrepancy in previous critical evaluations of charge radii.

S2. DATA ANALYSIS

The analysis of the extreme ultraviolet spectra focused on extracting the shift in Na-like $3p\,^2P_{1/2} \rightarrow 3s\,^2S_{1/2}$ and Mg-like $3s3p\,^1P_1 \rightarrow 3s^2\,^1S_0$ D_1 transition energies between naturally

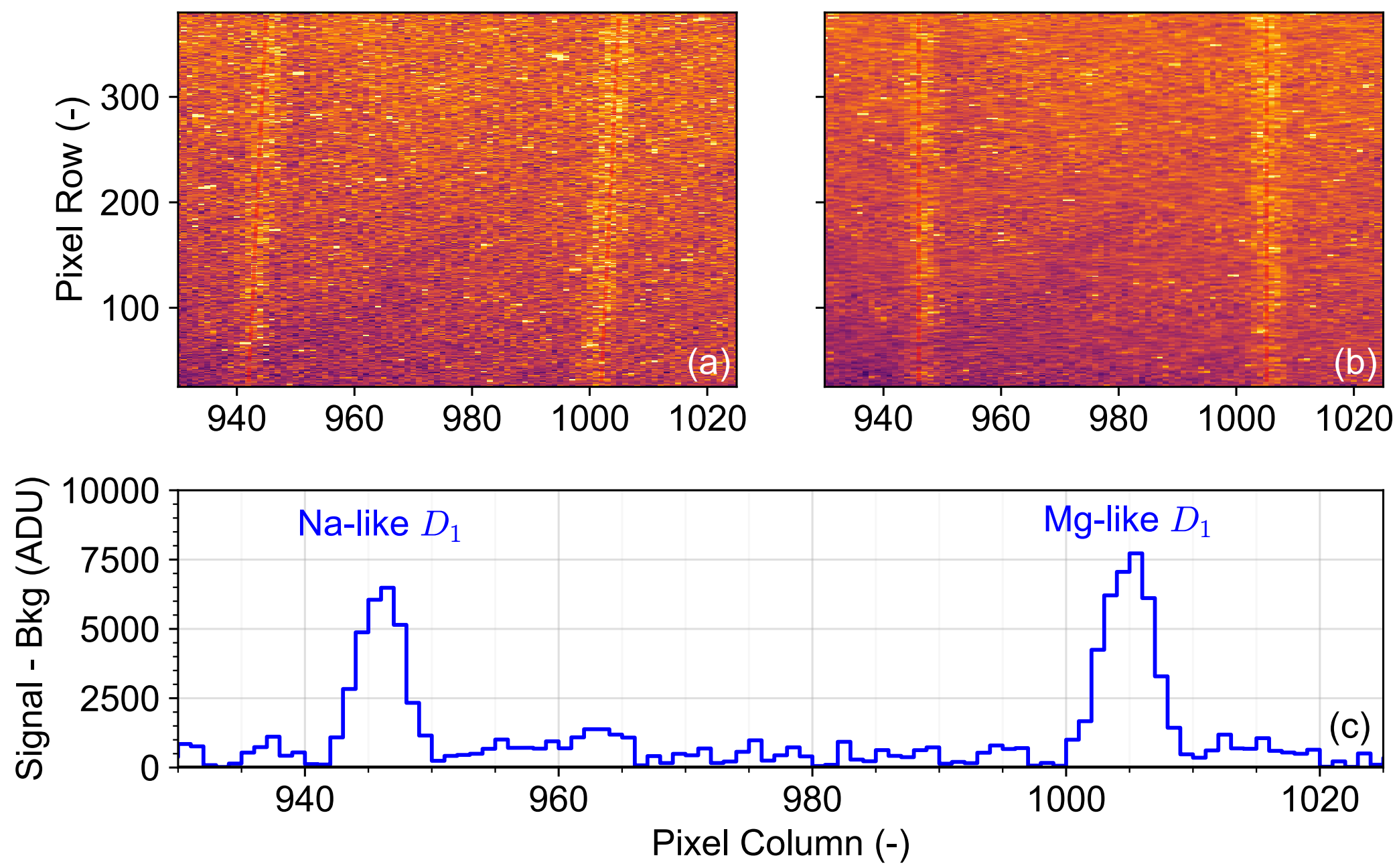

Supplementary Fig. 1. The processing steps of Sec. S2 A applied to a representative 50 minute Lu spectra. Images (a) and (b) represent the raw and processed images, respectively, with faint dashed red lines indicating the position of the Na-like and Mg-like D_1 line. Each column in (b) is then summed and then filtered to remove the flat-field background, with the collapsed spectra shown in (c).

abundant Lu and Yb ions. The data were processed using a combination of image correction, spectral fitting, and wavelength calibration techniques.

A. Spectral Processing and Corrections

Raw CCD images exhibited several systematic features, including slight tilting of spectral lines and a large background (Supplementary Fig. 1(a)). Line tilt was corrected by rotating the images by 0.8°. Cosmic ray artifacts were removed using contour detection algorithms [S13], ensuring clean spectra for subsequent analysis (Supplementary Fig. 1(b)). To address the time-varying background, we implemented a flat-field correction technique based on a sliding minimum filter. For each pixel, we applied a minimum value filter with a width of 20 pixels, much greater than the line width. The result was then passed through a low pass filter and Gaussian smoothing filter, and was then subtracted from the original spectra. The process from a raw image to a collapsed spectrum is shown in Supplementary Fig. 1.

B. Line Fitting and Calibration

Each 10-minute spectrum was analyzed individually. Spectral lines were fit using a Gaussian profile superimposed on a constant background, typically within a narrow window of

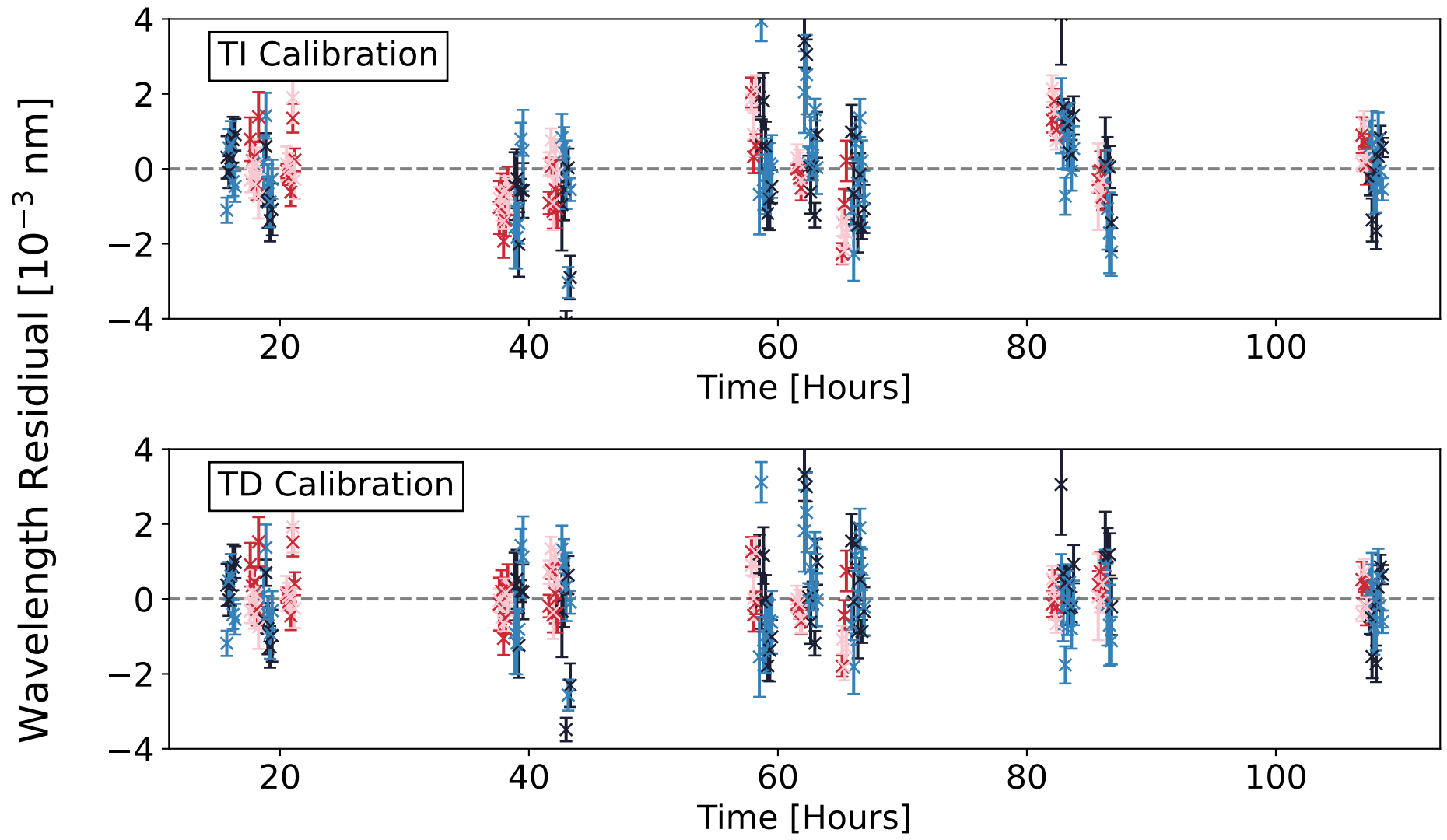

Supplementary Fig. 2. Comparison of the wavelength residuals from a time independent calibration procedure (top) and a time dependent calibration procedure (bottom). The Lu Na-like D_1 and Mg-like D_1 residuals are shown in dark blue and blue, respectively, with the Yb Na-like and Mg-like residuals shown in red and pink, respectively. Including a linear time dependence over the course of a day removes the majority of the structure in the residuals.

±8 pixels around the peak. Calibration was initially performed using well-known Ne and W emission lines [S14–S17]. However, due to profile complexity and blending in certain transitions—such as Al-like W and Li-like Ne—these lines were excluded from the final calibration fits.

A time-independent calibration model, based on a quadratic function in pixel space, yielded a reduced χ^2 of 3.2. We attributed this high reduced χ^2 to a small, but visually obvious time dependence of wavelength residuals in Supplementary Fig. 2, potentially coming from slight shifts in the electron beam position. To improve accuracy, a more refined time-dependent calibration model was implemented which allowed for a linear drift in time over the course of each day, but with a shared spatial dispersion for all days. To assist in determining the time drift, a term in the calibration loss function was added that penalized spread in the calibrated wavelengths of the Na- and Mg-like transitions of Lu and Yb; even though these wavelengths are unknown, they should be consistent over time. The time dependent procedure resulted in a reduced χ^2 of 2.4.

An F-test [S18] comparing the time-independent and time-dependent calibrations showed that the reduction in reduced χ^2 was statistically significant and could not be attributed solely to the additional parameters introduced by the time-dependent model. Increasing the time dependence beyond linear (e.g., quadratic or cubic terms) did not yield a statistically meaningful improvement in fit quality. Therefore, a linear time dependence was adopted.

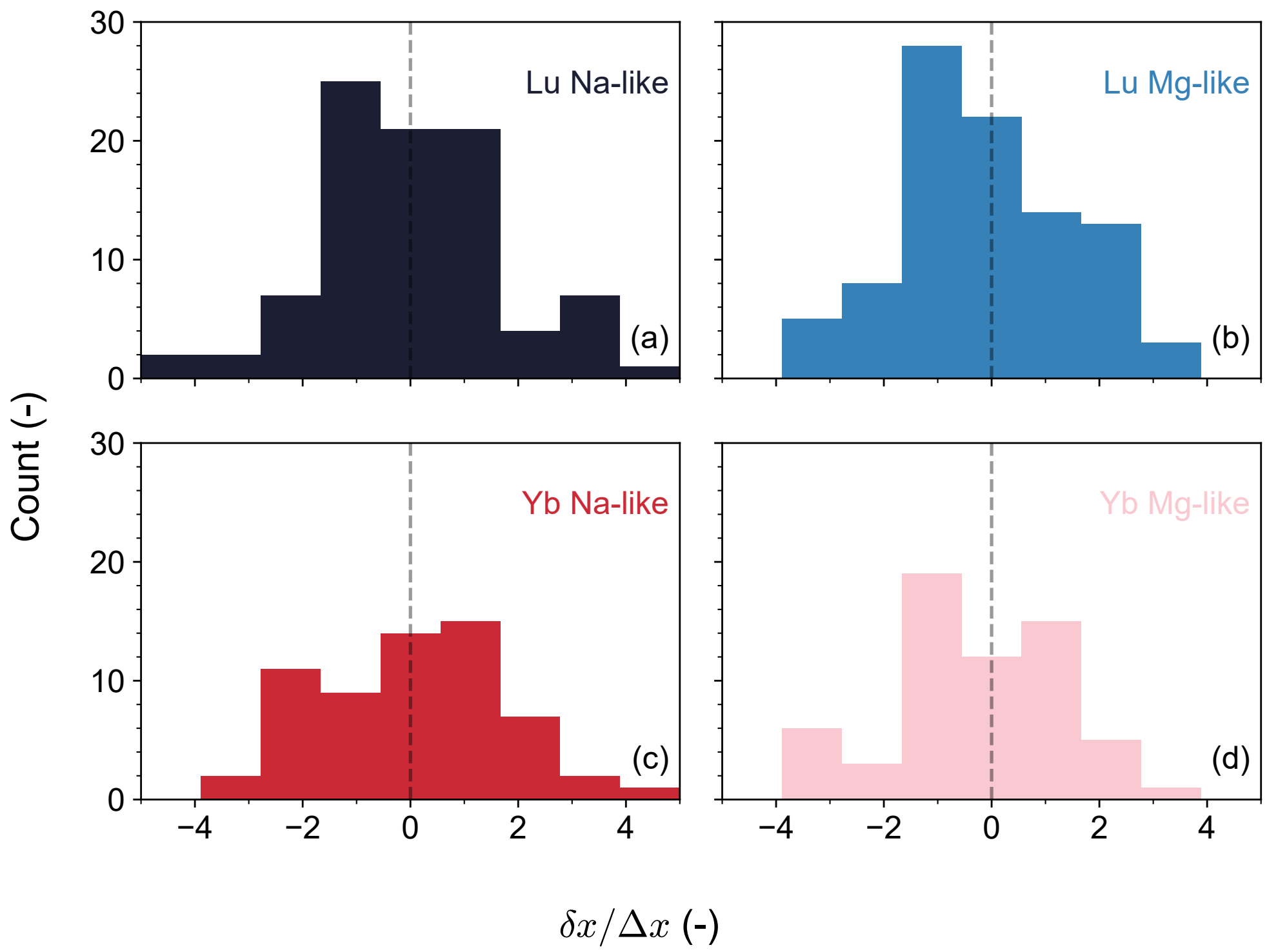

Supplementary Fig. 3. Histograms of the normalized residuals of the line positions in pixel space for all spectra. Panels show (a) Lu Na-like, (b) Lu Mg-like, (c) Yb Na-like, and (d) Yb Mg-like lines. For each roughly two-hour group of Lu and Yb, individual line positions were subtracted from the mean line position, and then divided by the fit uncertainty. The grouping of line positions was done to remove the structure due to long-scale time dependence. The distribution of normalized residuals is significantly wider then the expected normal distribution with standard deviation equal to one.

C. Transition Energy Shift Extraction

The remaining time-dependent reduced $\chi^2 = 2.4$ is indicative of a spread of line positions larger than what is suggested by the fitting uncertainty, as shown in Supplementary Fig. 3. Visual inspection of the line profiles did not reveal any obvious abnormalities, and processed images appeared free of any cosmic ray residuals that could have skewed line positions. This spread in the distribution could be symptomatic of small, short-timescale variations in line position that were too rapid to correct with our time-dependent calibration procedure. To address this apparent systematic underestimation of fitted line position uncertainties and the large reduced chi-squared, we have multiplied all experimental uncertainties by the square root of our calibration reduced chi-squared (the “Birge ratio”) [S19].

Using the time-dependent calibration and the fitted line positions, the Lu-Yb D_1 transition energy shifts were extracted for both Na-like and Mg-like charge states. Both the calibration of the spectrometer and the line statistics contribute to the experimental shift uncertainties.

S3. ATOMIC-STRUCTURE THEORY

A. Transition energies of Na- and Mg-like ions

Transition energies for Na-like ions are calculated using a method [S20, S21] that combines third-order relativistic many body perturbation theory (RMBPT) [S22] with first-principles QED corrections [S20]. As discussed in Ref. S21, various heuristics are employed to estimate omitted QED and many-body contributions, as well as the effects of methodological approximations in some of the terms. Numerical uncertainties arising from grid discretization, basis-set truncation, and integration accuracy are also assessed and are generally found to be negligible. A theoretical uncertainty is then assigned to each transition energy by combining all such effects. This approach yields excellent agreement with experimental D_1 transition energies across the whole Na-like isoelectronic sequence [S21, S23], suggesting that the estimated uncertainties—largely unchanged since the early work of Ref. S20, when experimental data were sparse—remain reliable indicators of the actual errors. Alternative approaches for the electronic structure of Na-like ions, including multiconfiguration Hartree-Fock calculations [S24] and an S-matrix QED approach [S25], can also be used and produce consistent results for the extracted charge radii [S24].

In contrast, the atomic structure of Mg-like ions, which have two valence electrons, is significantly more complex. The $n = 3$ valence complex exhibits strong configuration interaction, rendering a straightforward order-by-order RMBPT expansion ineffective. To address this, we adapt a strategy originally developed for Zn-like ions [S26], which face analogous issues in the $n = 4$ complex. An effective Hamiltonian for the $n = 3$ valence manifold is constructed using third-order RMBPT, supplemented by approximate *ab initio* QED corrections computed in a similar way to those for Na-like ions. The energies of Mg-like states are then obtained by diagonalizing this Hamiltonian. The effective Hamiltonian includes *one-body* terms describing valence-core interactions and *two-body* terms accounting for valence-valence interactions. The one-body terms also occur in the theory of Na-like ions, and the effective Hamiltonian can be conveniently organized so that Na-like transition energies appear along the diagonal. The two-body terms, which are specific to Mg-like ions, contribute to both the diagonal and off-diagonal terms. Zero-body contributions are also present, corresponding to the Ne-like core energy, but these cancel in transition energies and are therefore omitted, reducing the computational complexity substantially. This hybrid CI-RMBPT framework [S26] has not yet been applied systematically across the Mg-like isoelectronic series, but very good agreement is found for the Mg-like D_1 transition in Yb (see Supplementary Table I), with an estimated theoretical uncertainty only slightly larger than for the corresponding Na-like D_1 transition.

Theoretical D_1 transition energies for Na- and Mg-like Yb and Lu are summarized in Supplementary Table I. Contributions are separated into RMBPT orders $E(0)$–$E(3)$, where the zeroth-order term $E(0)$ corresponds to the Breit-Coulomb Dirac-Fock (BC-DF) approximation for the Ne-like core, and higher orders of RMBPT also incorporate both Coulomb and Breit interactions. Na-like ions involve only one-body terms, whereas Mg-like ions include both one- and two-body contributions. The QED sector includes valence self-energy, Uehling, and Wichmann-Kroll corrections, as well as approximate treatments of valence-exchange and core-relaxation QED terms. Two-loop QED contributions are incorporated

Supplementary Table I. Theoretical D_1 transition energies of Na- and Mg-like Yb and Lu. The last two columns give the difference of the transition energies (Lu minus Yb). Estimated theoretical uncertainties are shown in parentheses (when different from zero). Notation: “1-body”, core-valence terms; “2-body”, valence-valence terms; “E(0)”–“E(3)”, orders 0–3 of RMBPT including the Coulomb and Breit interactions; “1body-ret” and “2body-ret”, retardation terms; “Nuc.Rec.”, nuclear-recoil terms; “Nuc.Pol.”, nuclear-polarization terms; “Hyperfine“, hyperfine effect shift; “SE(val)”, valence self-energy; “Uehl(val)”, valence Uehling term; “WK(val)”, valence Wichmann-Kroll term; “QED(val-x)”, valence-exchange QED (self-energy plus vacuum polarization); “QED(core)”, core-relaxation QED; “QED(2-loop)”, phenomenological two-loop QED; “QED(2-body)”, valence-valence QED in Mg-like ions. Units: eV.

Contribution	Yb[a] ($Z=70$)		Lu[b] ($Z=71$)		Lu − Yb	
	Na-like D_1	Mg-like D_1	Na-like D_1	Mg-like D_1	Na-like D_1	Mg-like D_1
E(0)	150.649	150.649	154.075	154.075	3.4258	3.4258
E(1)		−4.618		−4.622		−0.0043
E(2,1-body)	−0.317	−0.311	−0.321	−0.316	−0.0042	−0.0042
E(2,2-body)		0.043		0.039		−0.0032
E(3)	0.004	0.004	0.004	0.004	0.0001	0.0001
1body-ret	0.002(8)	0.000(8)	0.004(9)	0.002(9)	0.0021(3)	0.0021(3)
2body-ret		0.000		0.000		0.0000
Nuc.Rec.	−0.007(1)	−0.007(1)	−0.007(1)	−0.007(1)	0.0000(1)	0.0000(1)
Nuc.Pol.	0.001(1)	0.001(1)	0.001(1)	0.001(1)	0.0000(5)	0.0000(5)
Hyperfine	0.000	0.000	0.000	0.000	0.0000	0.0000
SE(val)	−4.632	−4.562	−4.895	−4.822	−0.2624	−0.2597
Uehl(val)	0.762	0.750	0.818	0.806	0.0566	0.0559
WK(val)	−0.026(2)	−0.026(2)	−0.029(2)	−0.028(2)	−0.0026(2)	−0.0025(2)
QED(val-x)	0.057(4)	0.056(4)	0.060(4)	0.059(4)	0.0025(2)	0.0025(2)
QED(core)	−0.097(7)	−0.096(7)	−0.102(7)	−0.100(7)	−0.0044(3)	−0.0044(3)
QED(2-loop)	0.012(4)	0.012(4)	0.013(4)	0.013(4)	0.0009(3)	0.0009(3)
QED(2-body)		0.040(12)		0.041(12)		0.0014(4)
Total	146.407(13)	141.936(17)	149.622(13)	145.146(18)	3.2144(8)	3.2103(9)
Experiment	146.399(9)[c]	141.938(5)[c]				

[a] Nuclear parameters: $R = 5.3051$ fm, $\beta_2 = 0.320$, $t = 2.3$ fm (see Sec. S4).
[b] Nuclear parameters: $R = 5.3701$ fm, $\beta_2 = 0.305$, $t = 2.3$ fm (see Sec. S4).
[c] Reference [S27]

phenomenologically. There are also two-body QED terms specific to Mg-like ions. Further details of the various terms are given in Refs. [S20–S22, S26].

We note that because the Mg-like transition-energy differences in Supplementary Table I are obtained by matrix diagonalization, it is not possible to define unique additive contributions such as $E(0)$, $E(1)$, $E(2)$, etc., that sum linearly to yield the final transition energy. Instead, we construct the effective Hamiltonian incrementally: terms are added one at a time, and the matrix is rediagonalized after each addition. The contribution of a given term is then defined as the change in the eigenvalues when that term is included.

It should be emphasized that the contributions obtained this way are not uniquely defined. Because the diagonalization is nonlinear, the value attributed to a term may vary slightly depending on the order in which terms are introduced into the effective Hamiltonian. The first term added should be $E(0)$, corresponding to differences of BC-DF valence eigenvalues, since in a perturbative treatment of the diagonalization these reappear as the familiar energy denominators of RMBPT. The ordering of the remaining terms is, in prin-

ciple, arbitrary; here we adopt the sequence shown in Supplementary Table I (from top to bottom). Importantly, the final total, in which all effects are included, is independent of the order in which the terms are added.

B. Uncertainty in theoretical transition energies

Most theoretical contributions to the transition energy are electronic in nature and vary smoothly as functions of the nuclear charge Z. However, nuclear effects can introduce small irregularities. The dominant such contribution is the nuclear-size correction, which we exploit in this work to constrain nuclear charge radii. Other nuclear effects include nuclear recoil, nuclear polarization, and the hyperfine interaction.

The recoil correction, which depends on the nuclear mass, is negligible for the present application, amounting to only 0.0000(1) eV in the Lu–Yb transition-energy difference (see Supplementary Table I). Regarding the hyperfine effect, if the hyperfine levels in the EBIT are populated statistically, any hyperfine shift is expected to average to zero at the line center. In contrast, a non-statistical population can introduce asymmetries in the line shape and cause a small shift in the measured energy, as can second-order hyperfine shifts. We estimate both effects to be below 0.1 meV in the transition-energy difference, rendering them entirely negligible.

Among the other nuclear effects, the most uncertain contribution is the scalar nuclear-polarization (NP) shift. As an example of an explicit estimate of this shift, we apply the Coulomb-approximation method of Refs. S28 and S29, with the effective polarization potential of Ref. S29 included in the BC-DF procedure. We take the A-dependent fit to the nuclear electric-dipole (E1) polarizability $\alpha^{E1}(A)$ from Ref. S30, and assume the approximate formula $B(E2) = (3/4\pi)^2 Z^2 e^2 c_{\rm nuc}^4 \beta_2^2$ for the reduced electric-quadrupole (E2) transition probability to the first-excited collective rotational state [S31], where $c_{\rm nuc}$ is the half-density nuclear radius and β_2 is the quadrupole deformation. For the Na-like D_1 transition in ^{174}Yb, using the nuclear data in Supplementary Table IV, this procedure gives an NP-E1 shift of 0.23 meV and an NP-E2 shift of 0.33 meV, for a total NP shift of 0.56 meV.

In Sec. S4, we use the natural-abundance-averaged Lu-Yb transition-energy difference to extract the scaled-radius difference. In this Lu-Yb difference, the NP contribution is expected to cancel systematically to some extent. If we apply the method described above to the stable Lu and Yb isotopes, we obtain an NP difference of $\delta(\mathrm{NP}) \approx 0.04$ meV. This procedure likely overestimates the NP cancellation, however, because the relevant nuclear parameters may vary somewhat irregularly between neighboring nuclei, rather than follow the smooth parametrizations assumed here. Also, the formula for $B(E2)$ strictly applies only to $0^+ \to 2^+$ transitions in even-even nuclei. We therefore assign a theoretical uncertainty to $\delta(\mathrm{NP})$ that is a significant fraction of the NP shift itself. In addition, Refs. S32 and S33, which use nuclear density-functional theory (DFT), suggest that including excitations to additional nuclear states, together with transverse-photon terms, can increase the NP shift by roughly a factor of two in this range of Z. Such DFT calculations have not yet been performed for the highly deformed Lu or Yb isotopes. Nevertheless, even after accounting for these considerations, it is reasonable to conclude that the theoretical uncertainty in $\delta(\mathrm{NP})$ enters at the sub-meV level. In Supplementary Table I, we assign an uncertainty of 1 meV to the NP shift in a transition energy, and an uncertainty of 0.5 meV to $\delta(\mathrm{NP})$.

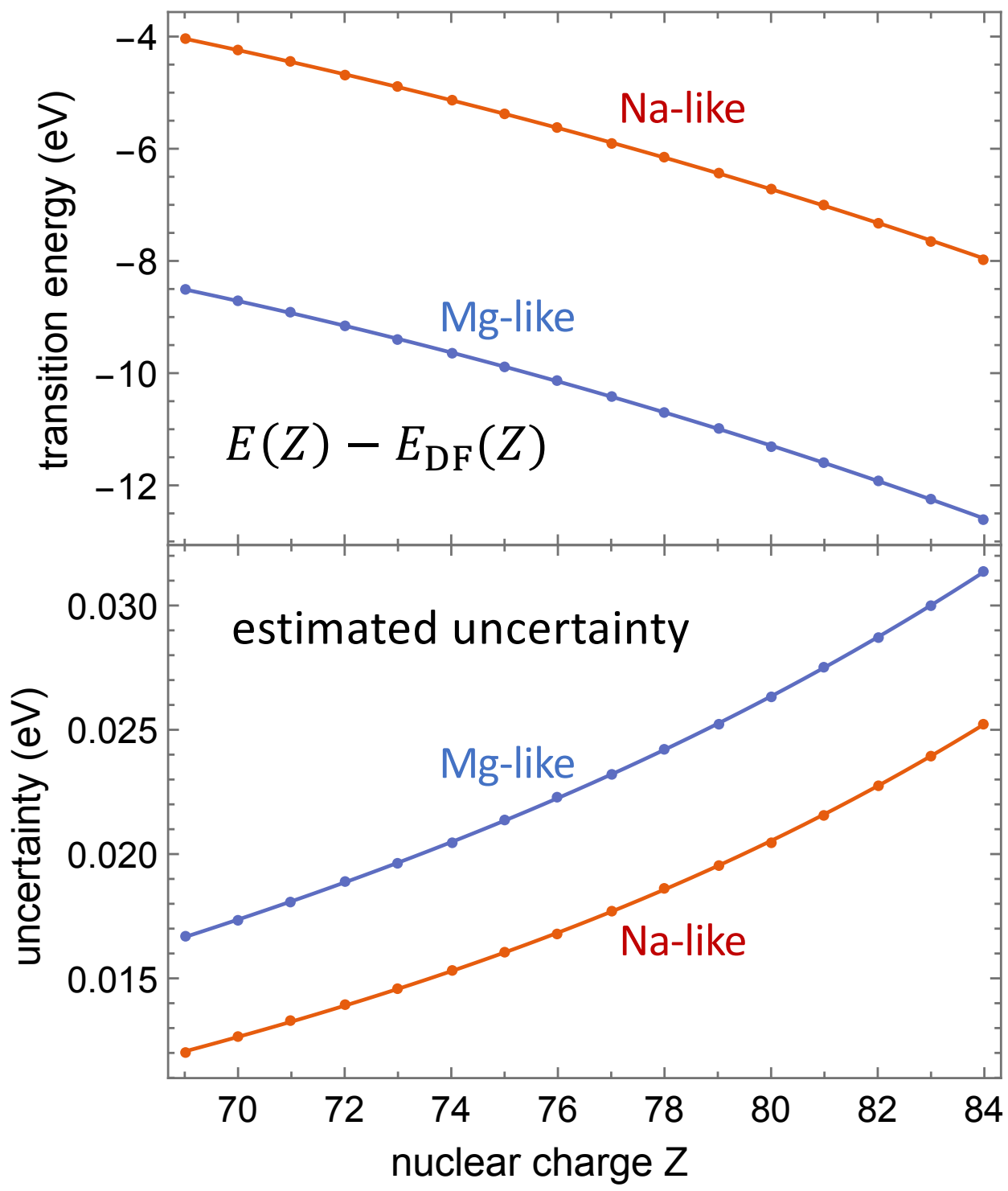

Supplementary Fig. 4. Top panel: Difference between the calculated D_1 transition energies $E(Z)$ and the corresponding Breit-Coulomb Dirac-Fock values $E_{\mathrm{DF}}(Z)$ for $69 \leq Z \leq 84$. Bottom panel: estimated theoretical uncertainties in the transition energies. Dots represent calculated values at each nuclear charge Z, and curves show polynomial fits to the data. Na-like D_1 transitions are shown in red, Mg-like D_1 transitions in blue.

The analysis in Sec. S4 B and Supplementary Table V shows that a 0.5 meV uncertainty in δ(NP) corresponds to only 3% of the total uncertainty budget. We therefore conclude that NP shifts are not significant for the present application, even if it turns out that we have slightly underestimated the true errors. This result highlights an important difference between the present EUV method and the muonic-atom approach, for which nuclear polarization (and deformation) can introduce significant uncertainties in this region of the nuclear chart.

In Supplementary Fig. 4, we show $E_\Delta(Z) = E(Z) - E_{\mathrm{DF}}(Z)$, the difference between the calculated D_1 transition energies $E(Z)$ and the corresponding Dirac-Fock baseline value $E_{\mathrm{DF}}(Z)$, which already incorporates the leading nuclear-size, recoil, and NP corrections. Irregularities in $E_\Delta(Z)$ as a function of Z may arise from residual nuclear-size effects, other residual nuclear contributions, or numerical uncertainties in the calculations. In practice, however, all such deviations are very small, and the data for $E_\Delta(Z)$ in Supplementary Fig. 4 are accurately described by a smooth polynomial fit, denoted $E_f(Z)$. Thus, the transition-energy correction $E_\Delta(Z)$ is principally an electronic quantity and largely free of irregularities associated with nuclear effects.

The true error in $E_\Delta(Z)$ is also dominated by uncalculated electronic terms and is therefore another highly smooth function of Z. Our quoted theoretical uncertainties on electronic-structure terms reflect estimates of these omitted higher-order terms. Because purely nu-

merical errors are negligible, these estimated uncertainties vary smoothly with Z and can be parameterized by smooth fitted functions $\Delta E_f(Z)$ (see Supplementary Fig. 4).

Now, if the true error in the calculated transition-energy correction $E_\Delta(Z)$ were given by some smooth function $\epsilon(Z)$, the error in the transition-energy difference $E_\Delta(Z) - E_\Delta(Z')$ would be simply $\epsilon(Z) - \epsilon(Z')$. Because $\epsilon(Z)$ is a smooth function, this error is systematic in nature. Noting that the estimated uncertainties $\Delta E_f(Z)$ reliably track the true error across the entire isoelectronic sequence, we therefore assume that $|\epsilon(Z)|$ can be approximated by $\Delta E_f(Z)$ and assign an uncertainty of $|\Delta E_f(Z) - \Delta E_f(Z')|$ to the calculated transition-energy difference. We can also obtain equivalent results, to within an accuracy of better than 0.1 meV, by making a simple two-point subtraction of the estimated uncertainties, $|\Delta E(Z) - \Delta E(Z')|$, without first finding the fitted functions $\Delta E_f(Z)$. Likewise, the computed transition-energy differences can be obtained equivalently from either $E_f(Z) - E_f(Z')$ or $E_\Delta(Z) - E_\Delta(Z')$, to better than 0.1 meV. This happens because the residual nuclear effects are small and our calculated electronic quantities are sufficiently free from potentially large random numerical effects. Using this subtractive procedure, we can assign uncertainties to all electronic-structure quantities in Supplementary Table I. The nuclear recoil, polarization, and hyperfine effects are assigned uncertainties separately, and all uncertainties are then added in quadrature. The resulting transition-energy differences between Lu and Yb, along with their estimated uncertainties, are listed in Supplementary Table I.

As can be seen from Supplementary Table I, this subtractive procedure generally yields *fractional* uncertainties in the transition-energy differences that are comparable to those of the individual transition energies. However, the *absolute* error in the transition-energy differences is strongly suppressed relative to that in the individual transition energies. Further discussion of these points is given in Section S3 C.

C. Suppression of uncertainty in transition-energy differences

In this section, we describe a simple way of understanding why the absolute uncertainty of the transition-energy difference is suppressed relative to that of the transition energy itself. Contributions to transition energies and their uncertainties can be approximated by polynomial fits of the form (e.g., see Supplementary Fig. 4)

$$P_n(Z) = a_n Z^n + a_{n-1} Z^{n-1} + a_{n-2} Z^{n-2} + \cdots \,, \tag{S1}$$

which may be interpreted as a leading term $a_n Z^n$ supplemented by correction terms in a $1/Z$ expansion.

In Sec. S3 B, we argue that when the error in the transition energy is a smooth, systematic function of Z (rather than random fluctuations), the uncertainty in the transition-energy difference is obtained by subtraction,

$$\begin{aligned} \Delta[E(Z) - E(Z')] &= |\Delta E(Z) - \Delta E(Z')| \\ &\approx |(Z - Z')\Delta E'(Z)| \,, \end{aligned}$$

where $\Delta E(Z)$ is the uncertainty in the transition energy $E(Z)$, and $\Delta E'(Z) = d[\Delta E(Z)]/dZ$. To illustrate this, suppose the uncertainty function $\Delta E(Z)$ of a particular contribution is

approximated by the leading term $a_n Z^n$ of the polynomial fit $P_n(Z)$ in Eq. (S1). In this case,

$$\frac{\Delta[E(Z)-E(Z')]}{\Delta E(Z)} \approx \frac{n}{Z}|Z-Z'|\,.$$

Thus, the uncertainty in the Lu-Yb transition-energy difference is naturally suppressed relative to the uncertainty in the corresponding transition-energy contribution by a factor n/Z.

The dominant omitted terms in the QED sector involve the treatment of vertex corrections in the contributions QED(val-x), QED(core), and QED(2-body) [S21, S26], which scale approximately as Z^3. Other errors in the QED sector associated with QED(2-loop) and WK(val) scale with higher powers of Z, as do errors in the treatment of retardation in "1body-ret" [S21]. Our total estimated uncertainty in the Z range of Supplementary Fig. 4 is found to scale approximately as $a_n Z^n$ with an effective index $n = n_{\text{eff}} \approx 3.5$. This is consistent with the suppression factor $n_{\text{eff}}/Z \approx 1/20$ found in this case (see Supplementary Table I).

Similarly, the fractional uncertainties in the transition energy $E(Z)$ and in the transition-energy difference $E(Z) - E(Z')$ are, respectively,

$$f_E = \frac{\Delta E(Z)}{E(Z)}\,, \qquad f_{\text{diff}} \approx \frac{\Delta E'(Z)}{E'(Z)}\,.$$

If $E(Z)$ and $\Delta E(Z)$ are represented by leading-order terms $a_m Z^m$ and $a_n Z^n$, respectively, then

$$\frac{f_{\text{diff}}}{f_E} \approx \frac{n}{m}\,,$$

which is of order unity. Hence, the fractional uncertainty in the transition-energy difference is generally comparable to that of the corresponding transition-energy contribution.

S4. NUCLEAR CHARGE RADII DETERMINATION

A. Scaled Charge Radius Difference

The extraction of cross-element charge-radius differences presented here extends the method recently applied to Ir using Na-like transitions [S24]. Here, we exploit the sensitivity of both Na-like ($3p\,^2P_{1/2} \to 3s\,^2S_{1/2}$) and Mg-like ($3s3p\,^3P_1 \to 3s^2\,^1S_0$) D_1 transitions to nuclear size effects, combining these measurements with high-precision atomic-structure calculations based on relativistic many-body perturbation theory and QED.

In the atomic structure calculations we assume a three-parameter Fermi nuclear charge distribution [S5] of the form

$$\rho(r,\theta,\phi) = \frac{\rho_0}{1+\exp\left[4\ln(3)(r-c_{\text{def}})/t\right]} \tag{S2}$$

with

$$c_{\text{def}}(\theta,\phi) = c[1+\beta_2 Y_{20}(\theta,\phi)]\,. \tag{S3}$$

The parameter t is the surface thickness, the distance over which the density falls from 90% to 10% of its central value. The parameter β_2 is the quadrupole deformation, with Y_{20} the

relevant spherical harmonic. The constant ρ_0 normalizes the distribution such that the total charge equals $Z|e|$. Varying the monopole half-density radius c allows adjustment of the *rms* charge radius, $R = \langle r^2 \rangle^{1/2}$.

In previous works [S24, S34], we handled the effect of higher order nuclear moments on charge radii determinations simply by inflating the uncertainty of the calculated nuclear sensitivity coefficient $S \equiv \partial E/\partial R$. We now present an improved formal description that better quantifies the uncertainty in extracted radii due to uncertainties in t and β_2.

Each choice of nuclear parameters produces a distinct theoretical transition energy, which we denote by $E^{\rm th} \equiv E^{\rm th}(R, t, \beta_2)$. For small deviations around a set of reference parameters (R_0, t_0, β_{20}), the theoretical energy can be approximated by a first-order Taylor expansion,

$$\begin{aligned} &E^{\rm th}(R, t, \beta_2) \\ &= E^{\rm th}(R_0, t_0, \beta_{20}) + \delta R \frac{\partial E}{\partial R} + \delta t \frac{\partial E}{\partial t} + \delta\beta_2 \frac{\partial E}{\partial \beta_2} \\ &\equiv E^{\rm th}(R_0, t_0, \beta_{20}) + S\delta R + S_t \delta t + S_{\beta_2}\delta\beta_2 \, , \end{aligned} \tag{S4}$$

where S, S_t, and S_{β_2} are nuclear *sensitivity* coefficients, defined as the partial derivatives of the transition energy with respect to R, t, and β_2, respectively. This linear approximation is valid for sufficiently small parameter variations. Second-order terms such as $\partial^2 E/\partial R^2$, $\partial^2 E/\partial R \partial t$, and $\partial^2 E/\partial R \partial \beta_2$ were evaluated and found negligible in this work (see Sec. S4 B).

As in previous works, we focus on transition-energy differences to take advantage of the resulting suppression in both experimental and theoretical uncertainties [S24, S34]. Equating theoretical and measured differences for two nuclides A and B yields

$$E^{\rm th}_B(R_B, t_B, \beta_{2B}) - E^{\rm th}_A(R_A, t_A, \beta_{2A}) = E^{\rm exp}_B - E^{\rm exp}_A \, , \tag{S5}$$

which, after substituting Eq. (S4), becomes

$$\begin{aligned} D_{BA} \equiv R_B &- (S_A/S_B) R_A \\ = &\,(E^{\rm exp}_B - E^{\rm exp}_A)/S_B \\ &- \big[E^{\rm th}_B(R_{0B}) - E^{\rm th}_A(R_{0A})\big]/S_B \\ &+ R_{0B} - (S_A/S_B) R_{0A} \\ &- (S_{tB}/S_B)(t_B - t_{0B}) \\ &+ (S_{tA}/S_B)(t_A - t_{0A}) \\ &- (S_{\beta_2 B}/S_B)(\beta_{2B} - \beta_{20B}) \\ &+ (S_{\beta_2 A}/S_B)(\beta_{2A} - \beta_{20A}) \, , \end{aligned} \tag{S6}$$

where $E^{\rm th}_B(R_{0B}) \equiv E^{\rm th}_B(R_{0B}, t_{0B}, \beta_{20B})$. We refer to $D_{BA} \equiv R_B - (S_A/S_B)R_A$ as the *scaled* radius difference. Equation (S6) shows that it is the scaled difference, rather than the simple difference $R_B - R_A$, that is directly constrained by a measurement of $E^{\rm exp}_B - E^{\rm exp}_A$.

Within the linear approximation of Eq. (S4), an equation of the same form as Eq. (S6) also holds for two *elements* A and B if all nuclear parameters (R, t, and β_2) are replaced by their corresponding isotopic-abundance average. For example, $R_A \to \bar{R}_A = \sum_i w_i R_{iA}$, where w_i denotes the isotopic abundance of the ith isotope of A with radius R_{iA}. This follows from abundance-averaging each side of Eq. (S6) with respect to A and B for constant

reference parameters $(R_{0A}, t_{0A}, \beta_{20A})$ and $(R_{0B}, t_{0B}, \beta_{20B})$. [Alternatively, one can start by abundance-averaging Eq. (S4).] In this case, the reference parameters can be taken to be suitable estimates of the abundance-averaged parameters. Here, we assign $A \to {}^{\rm nat}$Yb and $B \to {}^{\rm nat}$Lu, exploiting the accurately known radii and abundances of natural Yb isotopes to determine the natural-abundance-averaged radius of Lu.

Assuming reasonable estimates of the abundance-averaged parameters $\bar{t}$ and $\bar{\beta}_2$ for A and B can be found, we can use these as the reference parameters t_0 and β_{20}, and the final four terms in Eq. (S6) can then be set to zero. These terms do however contribute to the total uncertainty budget. Propagating uncertainties from Eq. (S6) yields

$$\begin{aligned}\left[\Delta(R_B - \frac{S_A}{S_B}R_A)\right]^2 &= \left[\frac{\Delta\left(E_B^{\rm exp} - E_A^{\rm exp}\right)}{S_B}\right]^2 + \left\{\frac{\Delta[E_B^{\rm th}(R_{0B}) - E_A^{\rm th}(R_{0A})]}{S_B}\right\}^2 \\ &+ \left\{\frac{\Delta S_B}{S_B}[(R_B - R_{0B}) - (S_A/S_B)(R_A - R_{0A})]\right\}^2 \\ &+ \left(\frac{S_{\beta_2 B}\Delta\beta_{2B}}{S_B}\right)^2 + \left(\frac{S_{tB}\Delta t_B}{S_B}\right)^2 \\ &+ \left(\frac{S_{\beta_2 A}\Delta\beta_{2A}}{S_B}\right)^2 + \left(\frac{S_{tA}\Delta t_A}{S_B}\right)^2 . \end{aligned} \tag{S7}$$

The last four terms arise from the uncertainties in $\bar{t}$ and $\bar{\beta}_2$. The third term arises from the uncertainty in the sensitivity coefficient S_B (due to omitted terms and numerical error in the atomic theory), while the analogous terms arising from the uncertainties in S_t and S_{β_2} are negligible and have been omitted. The quantity $\Delta[E_B^{\rm th}(R_{0B}) - E_A^{\rm th}(R_{0A})]$ represents the uncertainty in the theoretical energy differences assuming fixed nuclear parameters (i.e., no uncertainty in R_0, t_0, and β_{20}). This uncertainty therefore arises solely from omitted higher-order terms and numerical errors in the theory, and corresponds to the values listed in Supplementary Table I and discussed in Sec. S3.

This framework enables systematic uncertainty propagation and provides a robust methodology for extracting nuclear charge radii from precise spectroscopic measurements.

B. Sensitivity to Nuclear Density Parameters

Radii extracted using the HCI technique are largely insensitive to variations in t and β_2 when the charge radius R is fixed. To illustrate this, we determined sensitivity coefficients by evaluating the theoretical transition energies for five values of each nuclear-density parameter centered on its reference value, followed by polynomial fitting. For the radius sensitivity coefficient S, the energies were calculated using the full theoretical framework (RMBPT plus QED). The other two coefficients, S_t and S_{β_2}, primarily affect only the uncertainty through Eq. (S7); for these, the simpler BC-DF level of theory was employed. The resulting sensitivity coefficients are summarized in Supplementary Table II.

As shown in Sec. S4 D, the contributions of t and β_2 to the total uncertainty are very small. This outcome is already suggested by the relative magnitudes of the sensitivities to R, t, and β_2 in Supplementary Table II, indicating that even under weak constraints on the latter parameters, the radius difference can still be determined with high precision.

Supplementary Table II. Sensitivity coefficients for the Na-like and Mg-like D_1 transitions. The radius sensitivity S was calculated at the full RMBPT+QED level, while the skin-thickness sensitivity S_t and the deformation sensitivity S_{β_2} were evaluated at the BC-DF level.

	Yb		Lu	
	Na-like	Mg-like	Na-like	Mg-like
S [eV/fm]	−0.224(1)	−0.218(1)	−0.250(1)	−0.243(1)
S_t [eV/fm]	0.0010	0.0010	0.0012	0.0012
S_{β_2} [eV]	0.0024	0.0024	0.0027	0.0027

Supplementary Table III. Variations in $S_{\mathrm{Yb}}^{\mathrm{Na}}$ calculated at the BC-DF level for different nuclear model parameters. Unless otherwise indicated, calculations assume $R = 5.3051\,\mathrm{fm}$, $\beta_2 = 0.32$, $t = 2.3\,\mathrm{fm}$.

	S [eV/fm]
	−0.224367
$t = 2.1\,\mathrm{fm}$	−0.224350
$t = 2.3\,\mathrm{fm}$	−0.224387
$\beta_2 = 0.30$	−0.224386
$\beta_2 = 0.34$	−0.224346

We also consider the second-order size correction to Eq. (S4), $\delta E = S\delta R + S_2(\delta R)^2$ (for $\delta t = \delta\beta_2 = 0$). From the five-point polynomial fit at BC–DF level, we obtain second-order sensitivity coefficients $S_2 = -0.015$ eVfm^{-2} for Yb and $S_2 = -0.016$ eVfm^{-2} for Lu. The final inferred shift in the Lu radius is $\delta R\ = R - R_0 \approx 0.08$ fm relative to the reference value (see Sec. S4 D), implying a second-order energy correction of $S_2(\delta R)^2 \approx 0.1$ meV, which is negligible for the present purposes. Another way of looking at this is to note that the error in our extraction of the scaled-radius correction for Lu, due to our neglecting the second-order shift, is $\delta R_2 \approx S_2(\delta R)^2/S \approx 0.0004$ fm, or $(\delta R_2)^2 \approx 0.2\ 10^{-6}$ fm^2. One sees from Supplementary Table V that this radius error represents a negligible fraction of the total uncertainty budget in the scaled-radius difference. Moreover, this error in the radius determination can always be reduced simply by choosing the expansion point R_0 closer to the final inferred radius R. However, it is already negligible for the choice of expansion point made here.

The second way in which the neglect of second-order shifts can affect our analysis is in the natural-abundance averaging of Eq. (S6), which is based on the linear assumption. The most critical case here is the large range of stable Yb isotopes between ^{168}Yb and ^{176}Yb, for which $\delta R \approx 0.05$ fm [S4], corresponding to a second-order energy shift $S_2(\delta R)^2 \approx 0.04$ meV. This is again completely negligible for the present purposes. We conclude that the linear approximation in Eq. (S4) introduces negligible error.

To investigate the effect of higher-order terms such as $\partial^2 E/\partial R\partial t$ and $\partial^2 E/\partial R\partial\beta_2$ in Eq. (S4), we evaluated S at different values of β_{20} and t_0. The results in Supplementary Table III reveal that S fluctuates near the 0.01% level, a negligible amount relative to the

0.5% assumed uncertainty in S due to omitted terms in the atomic-structure theory.

For completeness, the adopted abundance-averaged values of t and β_2 for all naturally abundant Yb and Lu isotopes are provided in Supplementary Table IV.

Supplementary Table IV. Adopted skin thicknesses, deformation parameters, natural abundances, and isotopic radius differences for the isotopes considered in this work. Skin thicknesses are conservative estimates based on data from other rare-earth elements [S35]. The natural-abundance deformation parameters are computed as abundance-weighted averages across all isotopes, assuming complete error correlation within each isotopic chain.

	t [fm]	β_2 [-]	Abundance[a]	R^2 [fm^2]
^{168}Yb		0.322(9)[b]	0.126%	$R^2_{^{176}\mathrm{Yb}} - 0.505(101)$[c]
^{170}Yb		0.326(4)[b]	3.02%	$R^2_{^{176}\mathrm{Yb}} - 0.357(71)$[c]
^{171}Yb		0.328(5)[d]	14.2%	$R^2_{^{176}\mathrm{Yb}} - 0.305(61)$[c]
^{172}Yb		0.330(2)[b]	21.8%	$R^2_{^{176}\mathrm{Yb}} - 0.218(44)$[c]
^{173}Yb		0.328(4)[d]	16.1%	$R^2_{^{176}\mathrm{Yb}} - 0.168(34)$[c]
^{174}Yb		0.325(2)[b]	31.9%	$R^2_{^{176}\mathrm{Yb}} - 0.107(21)$[c]
^{176}Yb		0.305(5)[b]	12.6%	
^{175}Lu		0.31(3)[e]	97.4%	
^{176}Lu		0.30(3)[e]	2.6%	$R^2_{^{175}\mathrm{Lu}} + 0.041(4)$[e]
^{nat}Yb	2.3(2)	0.320(3)		
^{nat}Lu	2.3(2)	0.305(30)		

[a] Reference [S36]
[b] Reference [S37, S38]
[c] Reference [S39]
[d] Interpolated from neighboring isotopes
[e] Reference [S40]

We note that the present uncertainty estimates do not account for any dependence on the assumed nuclear model beyond the three-parameter Fermi distribution, Eq. (S2). It is improbable that the actual charge distribution is perfectly described by a Fermi function, as model-independent electron-scattering data demonstrate [S12]. Nevertheless, Supplementary Table II makes clear that the dominant effect on the D_1 transition energies is the charge radius, rather than higher-order nuclear moments. While a fully model-independent determination of radius differences from HCI transitions will be pursued in future work, using microscopic charge densities taken from nuclear DFT, we consider it justified at the current level of precision to neglect nuclear-model dependence.

C. Charge Radii Optimization Procedure

Given a set of (potentially correlated) measurements with normally distributed errors, non-linear generalized least squares provides the optimal parameters and their uncertainties [S41]. For a set of N measurements with mean values $\vec{\mu}$ and covariance matrix Σ, the optimal set of radii ($\vec{R}$) are those which minimize

$$\begin{bmatrix} \mu_1 - Q_1(\vec{R}) \\ \dots \\ \mu_N - Q_N(\vec{R}) \end{bmatrix}^T \Sigma^{-1} \begin{bmatrix} \mu_1 - Q_1(\vec{R}) \\ \dots \\ \mu_N - Q_N(\vec{R}) \end{bmatrix}, \tag{S8}$$

where $Q_i(\vec{R})$ is the quantity determined by the ith measurement. For example, if $\vec{R} = \begin{bmatrix} R_{^{175}\mathrm{Lu}} & R_{^{174}\mathrm{Yb}} \end{bmatrix}^T$, then $Q(\vec{R})$ of an absolute measurement of the radius of ^{174}Yb would simply be $Q(\vec{R}) = \left(\vec{R}\right)_2 = R_{^{174}\mathrm{Yb}}$.

The strong correlation between nuclear charge radii from optical isotope shift measurements can be handled explicitly using this formalism. To first order, frequency shifts between isotopes A and A' can be calculated from

$$\delta\nu^{AA'} \approx F\delta\langle r^2\rangle^{AA'} + K\delta\mu^{AA'}, \tag{S9}$$

where F is the field shift coefficient, and K is the mass shift coefficient. Rearranging to solve for $\delta\langle r^2\rangle^{AA'} \equiv \langle r^2\rangle^{A} - \langle r^2\rangle^{A'}$, we have

$$\delta\langle r^2\rangle^{AA'} = \frac{\delta\nu^{AA'} - K\delta\mu^{AA'}}{F}. \tag{S10}$$

In general, the uncertainty in $\delta\langle r^2\rangle^{AA'}$ is dominated by theoretical uncertainties in the field and mass shift coefficients. In the case of heavy elements (where the mass shift is much smaller than the field shift) this means that the uncertainty of OIS radius differences comes almost entirely from the uncertainty in the shared scaling of the field shift coefficient. Indeed, both [S40] and [S39] recommend common scaling uncertainties of 10% and 20%, respectively. This common uncertainty factor s can be encoded in the covariance matrix of a set of N nuclear charge radius isotope shifts from optical measurements $\delta\langle r^2\rangle$ as

$$\Sigma_{\mathrm{OIS}} = s^2 \begin{bmatrix} \delta\langle r^2\rangle_1^2 & \dots & \delta\langle r^2\rangle_1\delta\langle r^2\rangle_N \\ \vdots & \ddots & \vdots \\ \delta\langle r^2\rangle_1\delta\langle r^2\rangle_N & \dots & \delta\langle r^2\rangle_N^2 \end{bmatrix} \tag{S11}$$

with the $\mu - Q(\vec{R})$ vector from equation S8 given by

$$\vec{\mu}_{\mathrm{OIS}} - \vec{Q}_{\mathrm{OIS}}(\vec{R}) = \begin{bmatrix} (R_1^2 - R_2^2) - \delta\langle r^2\rangle^{A_1A_2} \\ \vdots \\ (R_1^2 - R_N^2) - \delta\langle r^2\rangle^{A_1A_N} \end{bmatrix}. \tag{S12}$$

The highly charged ion measurements in this work (Eqs. S16 and S17) lead to

$$\vec{\mu}_{\mathrm{HCI}} - \vec{Q}_{\mathrm{HCI}}(\vec{R}) = \begin{bmatrix} 0.354\ \mathrm{fm} - \left(\bar{R}_{\mathrm{Lu}} - \frac{S_{\mathrm{Yb}}^{\mathrm{Na}}}{S_{\mathrm{Lu}}^{\mathrm{Na}}}\bar{R}_{\mathrm{Yb}}\right) \\ 0.547\ \mathrm{fm} - \left(\bar{R}_{\mathrm{Lu}} - \frac{S_{\mathrm{Yb}}^{\mathrm{Mg}}}{S_{\mathrm{Lu}}^{\mathrm{Mg}}}\bar{R}_{\mathrm{Yb}}\right) \end{bmatrix} \tag{S13}$$

and

$$\begin{aligned} \Sigma_{\mathrm{HCI}} &= \begin{bmatrix} (\Delta_{\mathrm{Na}})^2 & \rho\Delta_{\mathrm{Na}}\Delta_{\mathrm{Mg}} \\ \rho\Delta_{\mathrm{Na}}\Delta_{\mathrm{Mg}} & (\Delta_{\mathrm{Mg}})^2 \end{bmatrix} \\ &= \begin{bmatrix} 147.8 & 24.2 \\ 24.2 & 159.8 \end{bmatrix} \mathrm{fm}^2. \end{aligned} \tag{S14}$$

See Sec. S4 D for a description of the covariance calculation between the Na-like and Mg-like scaled radius differences.

Therefore, an absolute measurement for Yb $R_{\mathrm{Yb}} = \mu_{\mathrm{Yb}} \pm \Delta_{\mathrm{Yb}}$, optical nuclear charge radii isotope shifts, and our HCI constraints determine an optimal set of parameters from the minimization of

$$\begin{aligned}\vec{R}_{\mathrm{opt}} = \arg\min_{\vec{R}} \Bigg\{ & \left(\frac{\mu_{\mathrm{Yb}} - R_{\mathrm{Yb}}}{\Delta_{\mathrm{Yb}}}\right)^2 + \\ & \left(\vec{\mu}_{\mathrm{OIS}} - \vec{Q}_{\mathrm{OIS}}(\vec{R})\right)^T \Sigma_{\mathrm{OIS}}^{-1} \left(\vec{\mu}_{\mathrm{OIS}} - \vec{Q}_{\mathrm{OIS}}(\vec{R})\right) + \\ & \left(\vec{\mu}_{\mathrm{HCI}} - \vec{Q}_{\mathrm{HCI}}(\vec{R})\right)^T \Sigma_{\mathrm{HCI}}^{-1} \left(\vec{\mu}_{\mathrm{HCI}} - \vec{Q}_{\mathrm{HCI}}(\vec{R})\right) \Bigg\}.\end{aligned} \tag{S15}$$

D. Results

To evaluate the theoretical transition energies, we must choose nuclear reference parameters (R_0, t_0, β_{20}) for Yb and Lu. These parameters define the expansion point for the linearization in Eq. (S4). Within the linear approximation, their precise values are immaterial; a different choice would yield the same inferred scaled-radius difference, provided that the selected values are sufficiently close to the physical values so that higher-order terms remain negligible. Accordingly, there is no uncertainty associated with (R_0, t_0, β_{20}), as indicated in Eq. (S7).

For t_0 and β_{20}, we adopt the best available abundance-averaged estimates $\bar{t}$ and $\bar{\beta}_2$ (see Supplementary Table IV and the discussion in Sec. S4 B). For the charge-radius reference parameter R_0 of Yb and Lu, we use the natural-abundance average of the previously recommended radii, giving $R_{\mathrm{Yb},0} = 5.3051$ fm and $R_{\mathrm{Lu},0} = 5.3701$ fm. As shown in Sec. S4 B, second-order size effects (associated with $\partial^2 E/\partial R^2$) are negligible for this choice of expansion point, as required. Other second-order terms involving t_0 and β_{20} are also discussed in Sec. S4 B and are likewise shown to be negligible.

The abundance-averaged experimental energy differences we determined are:

$$\begin{aligned} E_{\mathrm{Lu}}^{M,\mathrm{Na}} - E_{\mathrm{Yb}}^{M,\mathrm{Na}} &= 3.235(3)\,\mathrm{eV} \\ E_{\mathrm{Lu}}^{M,\mathrm{Mg}} - E_{\mathrm{Yb}}^{M,\mathrm{Mg}} &= 3.229(3)\,\mathrm{eV}. \end{aligned}$$

The theoretical transition energy differences evaluated at the reference parameters were calculated to be:

$$\begin{aligned} E_{\mathrm{Lu}}^{T,\mathrm{Na}}(\bar{R}_{\mathrm{Lu}_0}) - E_{\mathrm{Yb}}^{T,\mathrm{Na}}(\bar{R}_{\mathrm{Yb}_0}) &= 3.2144(6)\,\mathrm{eV}, \\ E_{\mathrm{Lu}}^{T,\mathrm{Mg}}(\bar{R}_{\mathrm{Lu}_0}) - E_{\mathrm{Yb}}^{T,\mathrm{Mg}}(\bar{R}_{\mathrm{Yb}_0}) &= 3.2103(7)\,\mathrm{eV}. \end{aligned}$$

Inserting these into Eq. (S6) yields the following constraints on the Yb and Lu nuclear radii

$$\bar{D}_{\mathrm{Lu,Yb}}^{\mathrm{Na}} = \bar{R}_{\mathrm{Lu}} - \frac{S_{\mathrm{Yb}}^{\mathrm{Na}}}{S_{\mathrm{Lu}}^{\mathrm{Na}}} \bar{R}_{\mathrm{Yb}} = 0.535(12)\ \mathrm{fm} \tag{S16}$$

$$\bar{D}_{\mathrm{Lu,Yb}}^{\mathrm{Mg}} = \bar{R}_{\mathrm{Lu}} - \frac{S_{\mathrm{Yb}}^{\mathrm{Mg}}}{S_{\mathrm{Lu}}^{\mathrm{Mg}}} \bar{R}_{\mathrm{Yb}} = 0.540(13)\ \mathrm{fm}. \tag{S17}$$

Supplementary Table V. Error budget for the Na-like and Mg-like determinations of the Yb-Lu scaled radius differences [Eqs. (S16) and (S17)]. The center column indicates the correlation coefficient between the two determinations for that term. The quantities in the uncertainty budget correspond to the uncertainty terms on the right-hand side of Eq. (S7).

Variance Contribution	Na-like [10^{-6} fm^2]	$\rho_{\mathrm{Na,Mg}}$	Mg-like [10^{-6} fm^2]
Lu, Stat	64.3	0.00	69.0
Yb, Stat	56.7	0.00	61.2
Calibration	15.2	0.97	14.5
**Experiment Total**	136.3	0.10	144.7
$E_B^{\mathrm{th}}(R_{B_0}) - E_A^{\mathrm{th}}(R_{A_0})$	9.8	0.70	13.0
S	0.1	1.00	0.1
**Theory Total**	9.9	0.70	13.1
t_{Lu}	0.9	1.00	1.1
$\beta_{2,\mathrm{Lu}}$	0.1	1.00	0.1
t_{Yb}	0.6	1.00	0.7
$\beta_{2,\mathrm{Yb}}$	0.0	1.00	0.0
**Parameters Total**	1.6	1.00	1.9
**Total**	147.8	0.16	159.8

Supplementary Table VI. The ^{175}Lu radius and ^{175}Lu-^{174}Yb isotonic radius difference determined from this work using various sources of the Yb radii. Each row represents the combination of this work (Eqs. S16 and S17), the optical isotope shift measurements of [S40] and [S39], and different Yb radii. The optimization procedure to obtain the presented values is described in detail in Appendix S4 C. An13: [S4], Fr04: [S5], Ze75: [S6], Sa79 [S1].

Reference Radius [fm]	$R_{^{175}\mathrm{Lu}}$ [fm]	$R_{^{175}\mathrm{Lu}} - R_{^{174}\mathrm{Yb}}$ [fm]
An13: $\bar{R}_{\mathrm{Yb}} = 5.306(6)$	5.291(11)	−0.017(10)
Fr04: $\bar{R}_{\mathrm{Yb}} = 5.302(2)$	5.288(10)	−0.017(10)
Ze75: $R_{^{174}\mathrm{Yb}} = 5.308(6)$	5.290(11)	−0.017(10)
Sa79: $R_{^{174}\mathrm{Yb}} = 5.410(30)$	5.382(30)	−0.028(10)

According to our calculations, the ratios $S_{\mathrm{Yb}}^{\mathrm{Na}}/S_{\mathrm{Lu}}^{\mathrm{Na}}$ and $S_{\mathrm{Yb}}^{\mathrm{Mg}}/S_{\mathrm{Lu}}^{\mathrm{Mg}}$ differ from each other by less than one part in 10^4 (with $S_{\mathrm{Yb}}^{\mathrm{Na}}/S_{\mathrm{Lu}}^{\mathrm{Na}} = 0.896$). Therefore, Eqs. (S16) and (S17) imply that the Na- and Mg-like measurements yield mutually consistent Lu-Yb radius differences. The constraints in Eqs. (S16) and (S17) are illustrated in Fig. 1 of the main paper, along with all of the previously discussed muonic atom spectroscopy and elastic electron scattering results. Given the natural-abundance-averaged nature of our work, we converted previous measurements to their natural-abundance-averaged equivalent using the abundances of Ref. S36 and the optical isotope shift (OIS) studies of Ref. S40 and Ref. S39.

The uncertainty in $\bar{D}_{\mathrm{Lu,Yb}}^{\mathrm{Na}}$ and $\bar{D}_{\mathrm{Lu,Yb}}^{\mathrm{Mg}}$ is dominated by statistical uncertainties and resid-

ual calibration uncertainties, as shown by Supplementary Table V. The next largest source of uncertainty is the theoretical energy difference for fixed nuclear parameters (4%–5% of total variance), followed by the uncertainties associated with the skin thicknesses and deformation parameters (1% of total variance). $\bar{D}^{\mathrm{Na}}_{\mathrm{Lu,Yb}}$ and $\bar{D}^{\mathrm{Mg}}_{\mathrm{Lu,Yb}}$ are correlated with correlation coefficient $\rho = 0.15$. This correlation arises from the shared wavelength calibration (68% of total covariance), the correlated theoretical energy difference calculations (23% of total covariance) and the correlated non-radius nuclear parameters (8% of total covariance). The theoretical contribution to the covariance was determined by evaluating the uncertainty in the difference between the Na-like and Mg-like transition energies and extracting the covariance via $\mathrm{Cov}[A, B] = 1/2\,(\mathrm{Var}[A] + \mathrm{Var}[B] - \mathrm{Var}[A - B])$. The correlation between $\bar{D}^{\mathrm{Na}}_{\mathrm{Lu,Yb}}$ and $\bar{D}^{\mathrm{Mg}}_{\mathrm{Lu,Yb}}$ appears on the off-diagonal elements of Eq. (S14). The inclusion of this correlation increases the final uncertainties in the extracted radii and radius differences by approximately 10% relative to the uncorrelated case.

To interpret our result in terms of the radii of specific isotopes, a generalized least squares fit is necessary that considers the strong correlations present in the data. This was accomplished using a preliminary version of the `radbase` set of codes (available at github.com/hstaige), which treats the isotopic radii as parameters and optimizes them given a set of measurements, as described in Sec. S4 C. In addition to the constraints of Eqs. (S16) and (S17), we included the OIS radius differences of Ref. S40 for Lu, and those of Ref. S39 for Yb. For both OIS works, a high degree of correlation between the different radius differences was assumed, as the main source of uncertainty was the theoretical uncertainty in the field shift coefficient. The primary challenge of this analysis is selecting the measurement(s) to use for the absolute radius of individual Yb and Lu isotopes. We completed the optimization for various choices of included absolute measurements, and present the results in Supplementary Table VI.

We note that, although there is a breadth of OIS data available for Lu and Yb, any OIS results are sufficient for the purpose of determining the isotonic differences and absolute radii of the naturally abundant isotopes. The absolute uncertainties of OIS work are small enough that neglecting them entirely does not affect any of the results in this work by more than 20% of the reported uncertainty.

Supplementary Figures

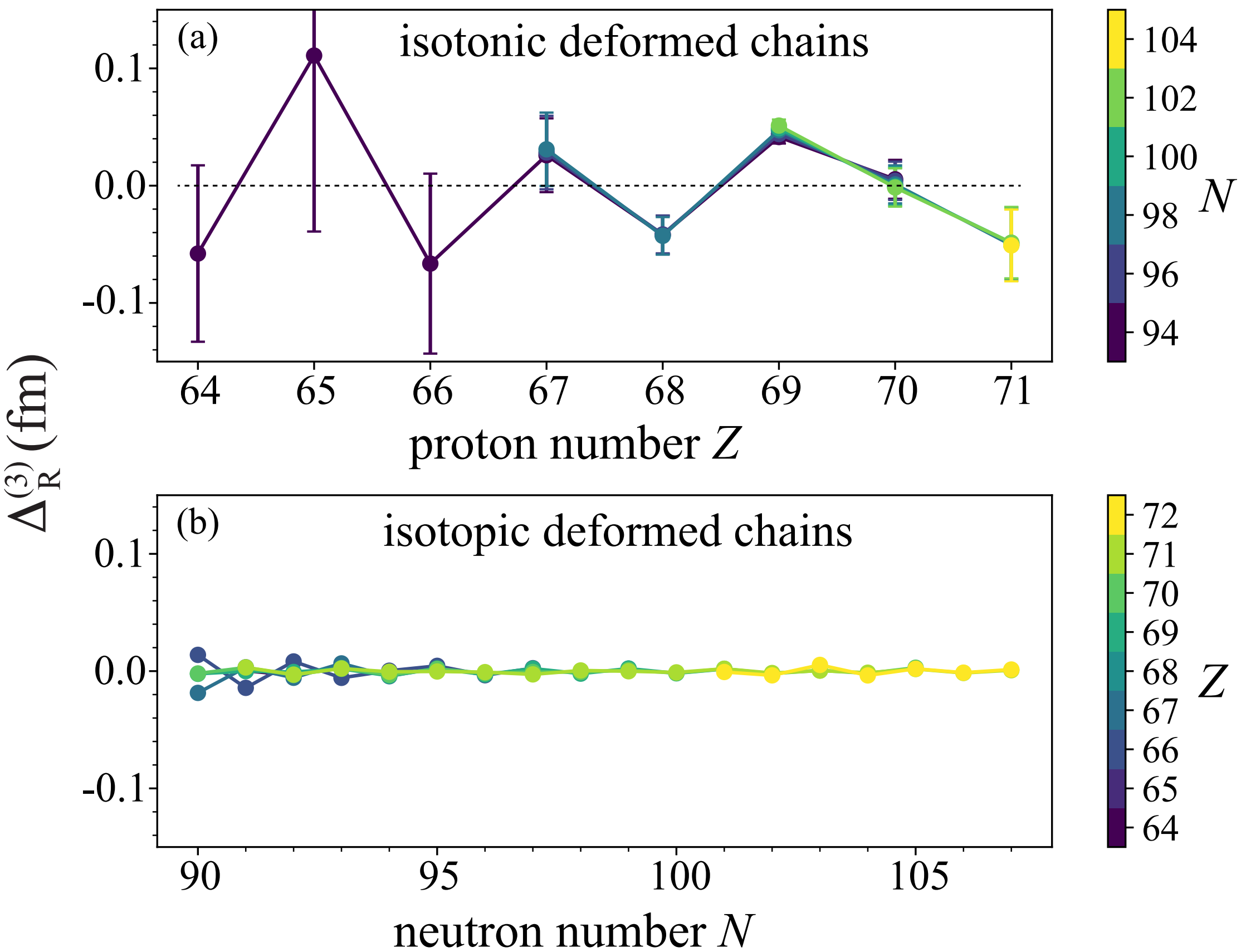

Supplementary Fig. 5. $\Delta_R^{(3)}$ of isotonic and isotopic chains of deformed nuclei calculated using recommended radii [S4]. (a) $\Delta_R^{(3)}$ along isotonic chains with even N. (b) $\Delta_R^{(3)}$ along isotopic chains. The isotonic stagger reported in this region is much larger than the isotopic stagger. The similarity of the different isotonic chains stems from each element's absolute radius being anchored by one or two measurements, creating a strong degree of correlation in the absolute radii between different isotopes of an element. Despite that, some elements have small enough uncertainty in their absolute radius for small differences between chains to be visible ($Z = 69, 70$, for example). Isotopic chains, on the other hand, are independent of each other, allowing for the different structure of each chain to be observed. Additional precision measurements of charge-radii in isotonic chains are needed to see whether the massive OESR seen in (a) is statistically significant, or whether it is a product of yet-uncontrolled systematic effects.

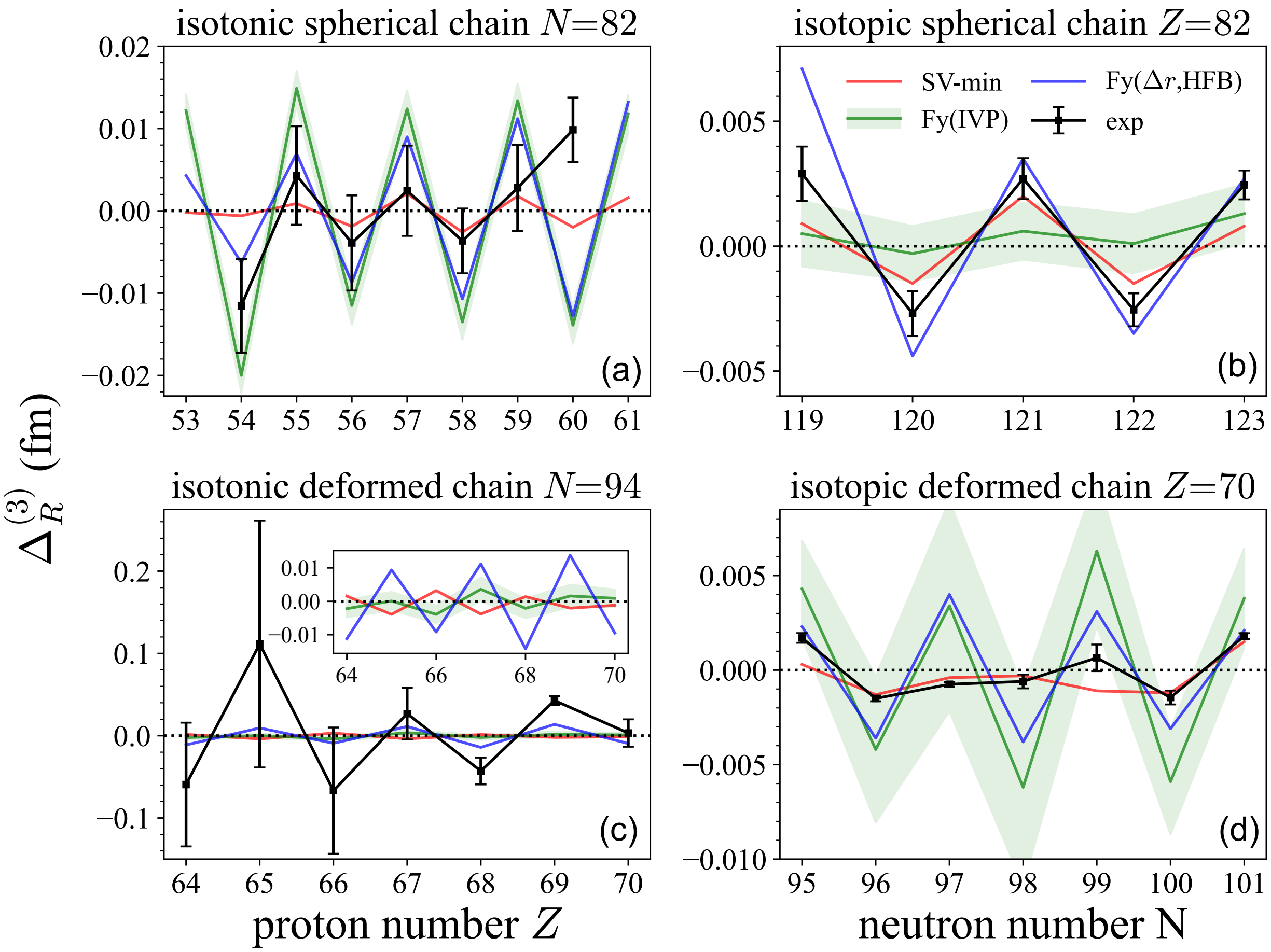

Supplementary Fig. 6. Similar to Fig. 4 in the main paper, but with each panel plotted in a different scale to better visualize trends and uncertainties: (a) isotonic spherical chain N=82, (b) isotopic spherical chain Z=82, (c) isotonic deformed chain N=94, and (d) isotopic deformed chain Z=70. Theoretical uncertainties are the sum of statistical uncertainties and a systematic uncertainty which includes the missing angular momentum projection for deformed nuclei. Uncertainties for different functionals are estimated to be similar.

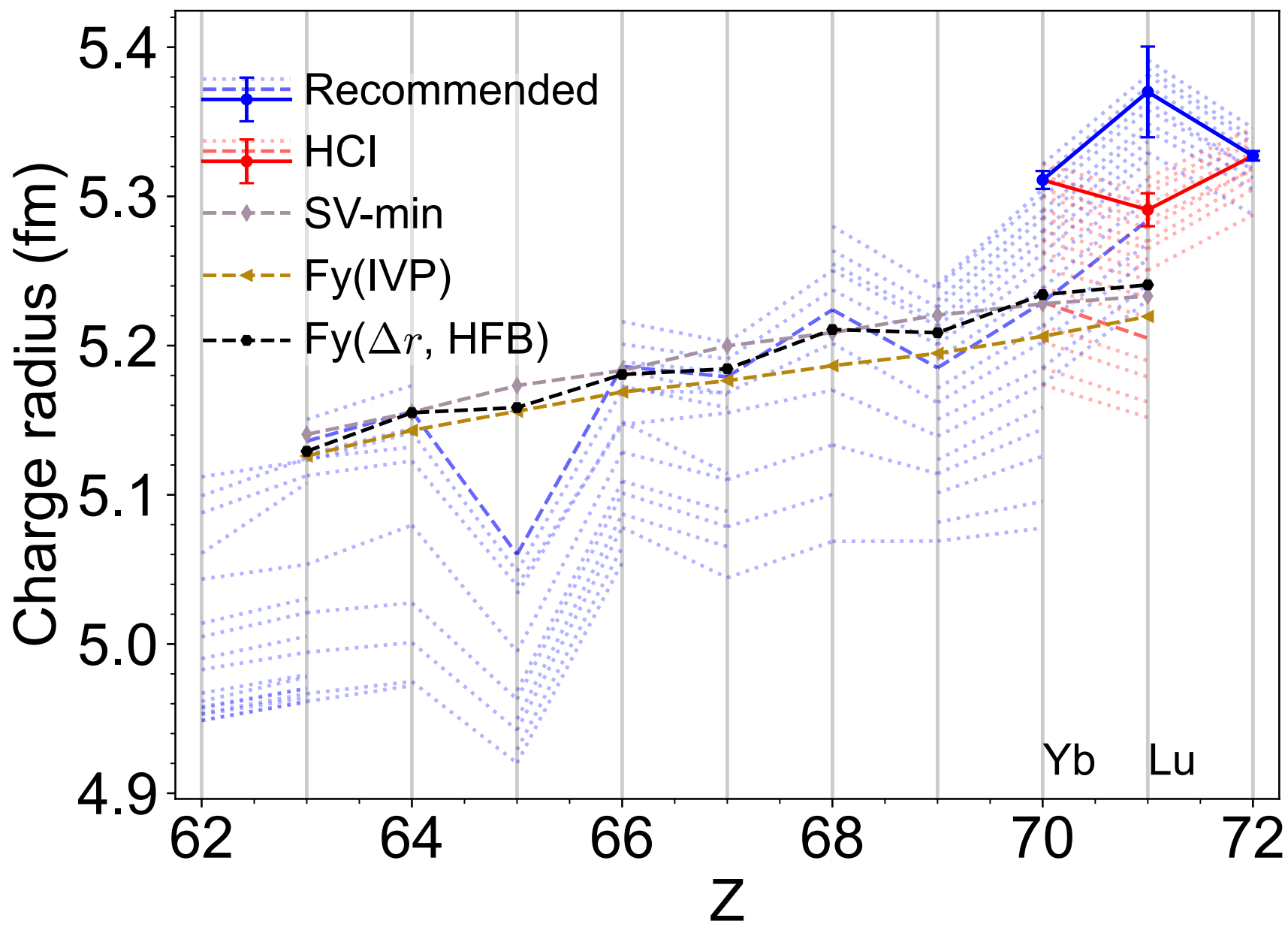

Supplementary Fig. 7. Restored odd-even staggering with the measured nuclear charge-radius difference between ytterbium and lutetium. The data points with solid blue lines and error bars correspond to the $N = 104$ isotonic sequence[S4] with our new result of $R_{^{175}\mathrm{Lu}} = 5.291(11)$ fm in red. Radii from Angeli and Marinova [S4] for the $N = 94$ isotonic chain are shown in dashed blue lines with the ^{165}Lu drop represented by the red dashed segment. Density functional theory predictions using functionals described in Methods/Nuclear Density Functional Theory Calculations are depicted by dashed black, brown, and gray lines. The series of blue dotted lines connect recommended values from Angeli and Marinova [S4], while the red dotted lines represent the OIS[S40] corrected values using our new anchor reference radius for ^{175}Lu.

Supplementary References

[S1] T. Sasanuma, *Electron Scattering from Deformed Heavy Nuclei*, Ph.D. thesis, Massachusetts Institute of Technology (1979).

[S2] T. Suzuki, *Muonic atom x-ray spectroscopy measurement of ^{175}Lu nuclear charge radius*, Ph.D. thesis, Carnegie Mellon University (1968).

[S3] R. Engfer, H. Schneuwly, J. Vuilleumier, H. Walter, and A. Zehnder, Charge-distribution parameters, isotope shifts, isomer shifts, and magnetic hyperfine constants from muonic atoms, At. Data Nucl. Data Tables **14**, 509 (1974).

[S4] I. Angeli and K. P. Marinova, Table of experimental nuclear ground state charge radii: An update, At. Data Nucl. Data Tables **99**, 69 (2013).

[S5] G. Fricke and K. Heilig, *Nuclear Charge Radii*, Landolt-Börnstein - Group I Elementary Particles, Nuclei and Atoms, Vol. 20 (Springer Materials).

[S6] A. Zehnder, F. Boehm, W. Dey, R. Engfer, H. Walter, and J. Vuilleumier, Charge parameters, isotope shifts, quadrupole moments, and nuclear excitation in muonic $^{170–174,176}$Yb, Nucl. Phys. A **254**, 315 (1975).

[S7] I. Angeli, *Table of Nuclear Root Mean Square Charge Radii*, Tech. Rep. INDC(HUN)-033 (IAEA Nuclear Data Section, 1999) available at `http://www-nds.iaea.or.at/indc-sel.html`.

[S8] D. T. Adler, *A study of the muonic x-rays from separated isotopes of ytterbium*, Ph.D. thesis, Carnegie Mellon University (1975).

[S9] C. Bernhardt, Ph.D. thesis, Universitat Mainz (1992).

[S10] T. Cooper, W. Bertozzi, J. Heisemberg, S. Kowalski, W. Turchinetz, C. Williamson, L. Cardman, S. Fivozinsky, J. Lightbody, and S. Penner, Shapes of deformed nuclei as determined by electron scattering: ^{152}Sm, ^{154}Sm, ^{166}Er, ^{176}Yb, ^{232}Th, and ^{238}U, Phys. Rev. C **13**, 1083 (1976).

[S11] C. W. Creswell, *Electron scattering studies of ^{166}Er, ^{176}Yb, and ^{238}U*, Ph.D. thesis, Massachutes Institute of Technology (1977).

[S12] H. De Vries and C. De Jager, C.W. De Vries, Nuclear charge-density-distribution parameters from elastic electron scattering, At. Data Nucl. Data Tables **36**, 495 (1987).

[S13] G. Bradski, The OpenCV Library, Dr. Dobb's Journal of Software Tools (2000).

[S14] A. Kramida and M.-C. Buchet-Poulizac, Energy levels and spectral lines of Ne VII, Eur. Phys. J. D **38**, 265 (2006).

[S15] A. Kramida and M.-C. Buchet-Poulizac, Energy levels and spectral lines of Ne VIII, Eur. Phys. J. D **39**, 173 (2006).

[S16] Y. Ralchenko, I. N. Draganic, J. N. Tan, J. D. Gillaspy, J. M. Pomeroy, J. Reader, U. Feldman, and G. E. Holland, EUV spectra of highly-charged ions W^{54+}–W^{63+} relevant to ITER diagnostics, J. Phys. B: At. Mol. Opt. Phys. **41**, 021003 (2008).

[S17] J. D. Gillaspy, I. N. Draganić, Y. Ralchenko, J. Reader, J. N. Tan, J. M. Pomeroy, and S. M. Brewer, Measurement of the D-line doublet in high-Z highly charged sodiumlike ions., Phys. Rev. A **80**, 010501(R) (2009).

[S18] D. B. Duncan, Multiple range and multiple F tests, Biometrics **11**, 1 (1955).

[S19] R. T. Birge, The calculation of errors by the method of least squares, Phys. Rev. **40**, 207

(1932).
[S20] S. A. Blundell, Calculations of the screened self-energy and vacuum polarization in Li-like, Na-like, and Cu-like ions, Phys. Rev. A **47**, 1790 (1993).
[S21] J. D. Gillaspy, D. Osin, Y. Ralchenko, J. Reader, and S. A. Blundell, Transition energies of the D lines in Na-like ions, Phys. Rev. A **87**, 062503 (2013).
[S22] W. R. Johnson, S. A. Blundell, and J. Sapirstein, Many-body perturbation-theory calculations of energy levels along the sodium isoelectronic sequence, Phys. Rev. A **38**, 2699 (1988).
[S23] H. Staiger, G. Mondeel, S. A. Blundell, Dipti, G. O'Neil, R. Silwal, A. Lapierre, G. Gwinner, J. N. Tan, J. D. Gillaspy, Y. Ralchenko, and E. Takacs, Measurement of D-line energies in sodiumlike Ir, Phys. Rev. A **112**, 012807 (2025).
[S24] A. Hosier, Dipti, S. A. Blundell, A. Lapierre, R. Silwal, G. Gwinner, J. N. Tan, A. Naing, J. D. Gillaspy, Y. Yang, *et al.*, Determination of nuclear charge radius by extreme-ultraviolet spectroscopy of Na-like ions, Phys. Rev. Res. **7**, L012024 (2025).
[S25] J. Sapirstein and K. T. Cheng, S-matrix calculations of energy levels of sodiumlike ions, Phys. Rev. A **91**, 062508 (2015).
[S26] S. A. Blundell, Calculation of QED corrections in highly charged Zn-like ions, Can. J. Phys. **87**, 55 (2009).
[S27] R. Silwal, Dipti, E. Takacs, J. M. Dreiling, S. C. Sanders, A. C. Gall, B. H. Rudramadevi, J. D. Gillaspy, and Y. Ralchenko, Spectroscopic analysis of M- and N-intrashell transitions in Co-like to Na-like Yb ions, J. Phys. B **54**, 245001 (2022).
[S28] A. V. Nefiodov, L. N. Labzowsky, G. Plunien, and G. Soff, Nuclear polarization effects in spectra of multicharged ions, Phys. Lett. A **222**, 227 (1996).
[S29] V. V. Flambaum, I. B. Samsonov, H. B. T. Tan, and A. V. Viatkina, Nuclear polarization effects in atoms and ions, Phys. Rev. A **103**, 032811 (2021).
[S30] M. Gorchtein, Guide to nuclear polarization in muonic atoms, Phys. Rev. C **113**, L011301 (2026).
[S31] A. Bohr and B. R. Mottelson, *Nuclear Structure, Vol. II: Nuclear Deformations* (World Scientific, Singapore, 1974).
[S32] A. Haga, Y. Horikawa, and Y. Tanaka, Nuclear polarization in hydrogenlike ${}^{208}_{82}Pb^{81+}$, Phys. Rev. A **65**, 052509 (2002).
[S33] I. A. Valuev and N. S. Oreshkina, Full leading-order nuclear polarization in highly charged ions, Phys. Rev. A **109**, 042811 (2024).
[S34] R. Silwal, A. Lapierre, J. D. Gillaspy, J. M. Dreiling, S. A. Blundell, Dipti, A. Borovik, G. Gwinner, A. C. C. Villari, Y. Ralchenko, and E. Takacs, Determination of the isotopic change in nuclear charge radius from extreme-ultraviolet spectroscopy of highly charged ions of Xe, Phys. Rev. A **101**, 062512 (2020).
[S35] G. Fricke, C. Bernhardt, K. Heilig, L. A. Schaller, L. Schellenberg, E. B. Shera, and C. W. Dejager, Nuclear ground state charge radii from electromagnetic interactions, At. Data Nucl. Data Tables **60**, 177 (1995).
[S36] M. Berglund and M. E. Wieser, Isotopic compositions of the elements 2009 (IUPAC Technical Report), Pure and Applied Chemistry **83**, 397 (2011).
[S37] S. Raman, C. W. N. Jr., and P. Tikkanen, Transition probability from the ground to the first-excited 2^+ state of even–even nuclides, At. Data Nucl. Data Tables **78**, 1 (2001).
[S38] B. Pritychenko, M. Birch, B. Singh, and M. Horoi, Tables of E2 transition probabilities from

the first 2^+ states in even–even nuclei, At. Data Nucl. Data Tables **107**, 1 (2016).

[S39] A. Kawasaki, T. Kobayashi, A. Nishiyama, T. Tanabe, and M. Yasuda, Isotope-shift analysis with the $4f^{14}6s^2\ {}^1s_0 - 4f^{13}5d6s^2\ (J = 2)$ transition in ytterbium, Phys. Rev. A **109**, 062806 (2024).

[S40] U. Georg, W. Borchers, M. Keim, A. Klein, P. Lievens, R. Neugart, M. Neuroth, P. M. Rao, C. Schulz, and the ISOLDE Collaboration, Laser spectroscopy investigation of the nuclear moments and radii of lutetium isotopes, Eur. Phys. J. A **3**, 225 (1998).

[S41] T. Kariya and H. Kurata, *Generalized Least Squares* (John Wiley & Sons, 2004).
