# Supplementary material for: Puzzling isotonic odd-even staggering of charge-radii in deformed rare earth nuclei
Source: Nat Commun. 2026 Sep 26;17:11159. doi: 10.1038/s41467-026-77471-7 (PMC13710950; doi:10.1038/s41467-026-77471-7)
Supplement: Supplementary file 2 [file 41467_2026_77471_MOESM2_ESM.pdf]

nature portfolio

Peer Review File

Puzzling Isotonic Odd-Even Staggering of Charge-radii in Deformed Rare Earth Nuclei

Corresponding Author: Professor Endre Takacs

This manuscript has been previously reviewed at another journal. This document only contains information relating to versions considered at Nature Communications.

This file contains all reviewer reports in order by version, followed by all author rebuttals in order by version.

Version 0:

Reviewer comments:

Reviewer #1

(Remarks to the Author)
In the revised version, the questions and comments raised in my previous report have been properly addressed. The extreme-ultraviolet spectroscopy of highly charged ions provides a unique opportunity to access the charge radii of nuclei that were previously not accessible. The revised manuscript presents more details on this new technique as well as the associated data analysis. Therefore, I consider the manuscript suitable for publication in Nature Communications in principle.

Before publication, I have one additional comment. As mentioned in the manuscript, the new technique provides precise measurements of charge-radius differences. However, the determination of absolute charge radii still relies on muonic atom data. As a result, the radii shown in Fig. 2(a) may inherit the limitations associated with charge-radius extraction in deformed nuclei due to their low-lying excitations. The authors should properly address this point in the final published version.

Reviewer #2

(Remarks to the Author)
Takacs and coworkers present in their “Puzzling Isotonic Odd-Even Staggering of Charge-radii in Deformed Rare Earth Nuclei” their combined experiments and theory efforts to successfully resolve the standing issue of the Lu inversion anomaly and reestablish the (now pronounced) odd-even staggering along the isotonic chain with neutron number N=94.

With the resolution of the long-standing Lu puzzle, the authors also introduce a new method of significance to probe odd-even staggering in charge-radii (OEST) as a function of proton number, across isotonic chains where data remains scarce due to experimental challenges that the EBIT spectroscopy, in tandem with calculations, appear to resolve.

The manuscript of particular high quality, and could be accepted for publication in its current form given the efforts of the authors to accommodate the suggestions and comments of the reviewer. In particular, it is appreciated that the manuscript is now more self-contained, that there is a more thorough presentation of the spectral data, and the risk of a logical fallacy is well explained and rebutted in the revised manuscript.

Reviewer #3

(Remarks to the Author)
This work represents an outstanding achievement at the intersection of atomic and nuclear physics experiment. The authors have innovatively developed extreme ultraviolet (EUV) spectroscopy of highly charged ions (such as Na-like and Mg-like ions) using the Tokyo Electron Beam Ion Trap into a high-precision, cross-element tool for comparing nuclear charge radii. The experimental design is ingenious: Lu and Yb atoms of natural isotopic abundance are alternately injected via Knudsen cells, ionized to high charge states under identical EBIT conditions, and their D1 lines are recorded with a flat-field grazing-

incidence spectrometer at a resolution of approximately 0.01 nm. To overcome spectrometer drift, the authors introduced calibration lines from W and Ne and established a time-dependent wavelength calibration model, thereby achieving stringent control over systematic errors.

The transition energy differences between Lu and Yb derived from the two independent spectral lines (Na-like and Mg-like) are compared with precise atomic structure calculations based on relativistic many-body perturbation theory combined with QED corrections. By solving the scaled radius difference equation, the authors determined the charge radius of ^{175}Lu to be 5.291(11) fm, representing a downward revision of approximately 0.08 fm from the previous recommended value. This result definitively proves that the nuclear charge radius of ^{175}Lu is smaller than that of ^{174}Yb, thereby resolving the decades-old "Lu inversion anomaly" and restoring the normal odd-even staggering pattern—where odd-proton nuclei are more compact—in the N=94 isotonic chain. This study provides a novel and precise probe for nuclear structure research. While it should be acknowledged that the authors and their research team have previously demonstrated aspects of this methodology in earlier publications, this does not diminish the significance of its application in the present work. On the contrary, the maturity and reliability of the technique, as evidenced by its prior development, lend greater confidence to the exceptional precision and robustness of the current cross-element measurement. The novelty here lies not in the first demonstration of the tool, but in its deployment to decisively solve a longstanding nuclear structure puzzle and to reveal a striking new anomaly, thereby fully realizing its potential as a transformative probe in nuclear physics.

Another highlight of the paper is the claim of discovering an "unexpectedly large" enhancement of odd-even staggering specific to the N=94 isotonic chain and the assertion that mainstream Density Functional Theory (DFT) "fails" in this region. Currently, this remains primarily a description of the phenomenon rather than a physical explanation. This finding is clearly worthy of further investigation.

In summary, this is a very solid piece of work, and the supplementary material provided by the authors is quite detailed. The authors have also addressed the previous reviewers' concerns with appropriate revisions and responses. That said, I acknowledge that the study's impact and depth may be viewed differently by some readers. Nevertheless, in my view, the manuscript is suitable for publication in NC.

Reviewer #4

(Remarks to the Author)

I would like to focus on the technical aspects of the manuscript. Despite the modifications made by Authors, the resubmitted manuscript still contains several noticeable issues:

1) There is a well-established terminology in the field, according to which the Authors have measured a *relative* radius. As can be seen from the reports of other referees, the terminology used in the manuscript is indeed confusing. I therefore recommend to revise it accordingly, and the results are not referred as "absolute" radii, but consistently as relative ones. E.g. Page 2, l.c:
"When anchored to muonic atom data for Yb [31], our result provides a new value of the absolute
nuclear charge-radii of Lu isotopes, including 175Lu" - here, absolute should be replaced by relative.
(The same part in the Authors' response reads as: "By combining our result with muonic-atom data, we infer a new value of the absolute nuclear charge-radii of Lu isotope..." - same problem).

2) It is still strongly recommended that uncertainties associated with the reference
radius be explicitly included in the uncertainty budget. For example, the reported value R[175Lu]=5.291(11)fm should be written as R[175Lu]=5.291(6)(10)fm, where the first set of parentheses reflects the uncertainty of R[174Yb]=5.3108(60)fm taken from Angeli (2013), and the second one correspond to the current work (in this case, the total uncertainty would effectively be 12 instead of 11, possibly a rounding issue).
2a) the reported value R[175Lu]=5.291(11)fm appears to be in disagreement with the value of 5.3700(304) reported in Angeli (2013). Any comments?

3) The added table S1 is very useful. It provides a much clearer understanding of which contributions are included in the calculations and which are not, and also raises some questions. Commonly, when extracting rms radii from isotope shifts, additional contributions should be analyzed, namely: higher-order term proportional to <r^2>^2, nuclear deformation <r^4>, higher-order nuclear recoil and nuclear polarizability. H.O. nuclear recoil is negligible here, as obvious from the first-order recoil contribution in the table. H.O. <r^2>^2 term is in some sense corresponds to my previously raised concern about the linearity across the considered range 94-104, and now somehow discussed. So,
3a) It would be desirable to evaluate this contribution explicitly, following the standard procedures used in isotope-shift analyses.
3b) Nuclear deformation is another important contribution. The Authors starts with a deformed Fermi distribution. How do they treat the deformation among the isotope chain? Is the same \beta_2 used through it, or for every isotope the value of deformation is different? In any of those cases, is the approach fully consistent and free from double-counting?
3c) finally, nuclear polarizability is currently fully omitted. A justification is required, especially since unlike all other QED effects, NP is highly nucleus-specific and can not be expected to fully cancel in the considered difference. Moreover, due to the large uncertainty of the effect itself, the remaining NP uncertainty in the isotonic difference can be orders of magnitude larger than the contribution itself. At minimum, estimates, clarifications, or justification should be provided.

4) A minor comment: Sup. Mat., page 9: "Regarding the hyperfine effect, if the hyperfine levels in the EBIT are populated statistically, any hyperfine shift is expected to average to zero at the line center." This hold only at first order, what about second-order hfs? How large is it compared to the claimed measurements accuracy?

5) The cancellation of uncertainties relies entirely on the assumption expressed in Eq. (S1), which itself is based on calculated-energies pattern. If certain contributions are missing, underestimated, or inaccurately computed, this approach would not reveal such deficiencies. I find the assumption (S1) over optimistic, and likely invalid over the considered range of Z=69-84. Even the energies themselves may exhibit a non-smooth behavior (e.g. due to NP contribution), and it is even more likely for the uncertainties (e.g. because of the different nuclear deformation parameter \beta_2 and its' uncertainty). I encourage the Authors to provide a convincing justification.

In light of the points raised above, I regret to conclude that the presented data currently do not seems to support the conclusions and claims, especially when it comes to the uncertainties. Several relevant contributions are either missing or insufficiently addressed in the theoretical analysis. The uncertainties budget is based on a seemingly empirical formula, which is not confirmed by a rigorous approach, likely leading to a significant underestimation of uncertainties. Therefore, the manuscript can not be published in its' present form.

Version 1:

Reviewer comments:

Reviewer #1

(Remarks to the Author)
In the revised version, my comments have been properly addressed, and I therefore recommend its publication now.

Reviewer #4

(Remarks to the Author)
The Authors have made an effort to address my previous comments. While most points have been answered, some responses remain somewhat superficial. Therefore, I must request further clarification and rigor regarding the following points before this manuscript can be considered for publication:

1) First and foremost, all discussed contributions must be explicitly included in Table S1, including nuclear polarizability and nuclear deformation, along with both their estimated values and associated uncertainties. Omitting these data from the table gives the incorrect impression that they have been ignored in the analysis.

2) "We have not explicitly estimated the nuclear-polarization contribution, which would require nuclear-structure calculations with considerable uncertainties. Recent work on nuclear polarization in the hydrogen-like 2s1/2 state of 208Pb found a correction below 10 meV [S28], and an order of magnitude smaller for the 3s1/2 state."
While I agree that an accurate calculation of NP is truly complex, a quantitative estimation must be provided. The reference to H-like systems is not entirely relevant here: because NP is a short-range interaction, core-excitation effects are highly significant. This means that the effect for an H-like 2s state can easily be an order of magnitude smaller than the NP effect for a Li-like system with an electron in the 2s state for the same nucleus. This effect is even more pronounced for 3s states. Additionally, it appears that the Authors have performed some preliminary estimations that are not shown in the manuscript. These estimations should be explicitly included.

3) "These results were subsequently used to estimate nuclear polarization in Li-like ions [S29], where it was shown to be comparable in magnitude to the nuclear-deformation effect, which exhibits a similar
Z scaling."
This statement is too vague, and more detailed information is required. What is the specific scaling being referenced? What are the calculated contributions for the reference systems? Most importantly, how do these translate into specific values for the HCIs measured in this work?

4) "In the Na-like and Mg-like systems considered here, both nuclear-polarization
and deformation effects are further suppressed."
This suppression holds true only if core-excitation effects are not-existing, which cannot be simply taken for granted.

5) "We find that the total nuclear-deformation contribution for the transitions studied here is at most 0.4 meV. "
Please justify the origin of this value. As currently written, this upper bound appears unsupported by data or citations.

Taking the 10 meV value cited by the Authors, scaling it up for core-excitation effects, and then down for the 3s state, it is entirely plausible that the final value of the NP contribution remains on the order of a few meV. According to the Authors', a similar value should be assigned to nuclear ND. Based on the justifications presented in the manuscript, I am not yet convinced that these contributions are truly negligible.

6) The correctness of the results is still being argued based on the assumption that the neglected nuclear effects are small. However, even if these neglected effects were significant, omitting them would likely still produce a smooth curve. Therefore, the smoothness does not serve as a valid justification for neglecting them.

All of these issues could likely be resolved by adopting a more conservative approach to the error estimations. Incorporating these uncertainties would preserve the impact of the work while ensuring it remains scientifically rigorous.

Based on the points raised above, I stay of the opinion that the underlying theory requires further refinement to robustly support the current claims. Consequently, I cannot recommend the manuscript for publication in its current form without these necessary improvements.

Version 2:

Reviewer comments:

Reviewer #4

(Remarks to the Author)
My comments are finally properly addressed, and the manuscript contains enough detail for reproducibility. I can now recommend the publication.

Open Access This Peer Review File is licensed under a Creative Commons Attribution 4.0 International License, which permits use, sharing, adaptation, distribution and reproduction in any medium or format, as long as you give appropriate credit to the original author(s) and the source, provide a link to the Creative Commons license, and indicate if changes were made.
In cases where reviewers are anonymous, credit should be given to 'Anonymous Referee' and the source.
The images or other third party material in this Peer Review File are included in the article's Creative Commons license, unless indicated otherwise in a credit line to the material. If material is not included in the article's Creative Commons license and your intended use is not permitted by statutory regulation or exceeds the permitted use, you will need to obtain permission directly from the copyright holder.
To view a copy of this license, visit https://creativecommons.org/licenses/by/4.0/

In what follows, we reproduce the reviewers' comments in blue and indicate our revisions in red. We also attach a PDF of the revised manuscript and SI with all changes marked in red.

Reviewer #1

In the revised version, the questions and comments raised in my previous report have been properly addressed. The extreme-ultraviolet spectroscopy of highly charged ions provides a unique opportunity to access the charge radii of nuclei that were previously not accessible. The revised manuscript presents more details on this new technique as well as the associated data analysis. Therefore, I consider the manuscript suitable for publication in Nature Communications in principle.

Before publication, I have one additional comment. As mentioned in the manuscript, the new technique provides precise measurements of charge-radius differences. However, the determination of absolute charge radii still relies on muonic atom data. As a result, the radii shown in Fig. 2(a) may inherit the limitations associated with charge-radius extraction in deformed nuclei due to their low-lying excitations. The authors should properly address this point in the final published version.

We thank the referee for this observation. We have clarified this point in the manuscript.

"These absolute values may inherit the limitations of the underlying reference, in this case those associated with charge-radius extraction from muonic-atom measurements in deformed nuclei."

Reviewer #2:

Takacs and coworkers present in their "Puzzling Isotonic Odd-Even Staggering of Charge-radii in Deformed Rare Earth Nuclei" their combined experiments and theory efforts to successfully resolve the standing issue of the Lu inversion anomaly and reestablish the (now pronounced) odd-even staggering along the isotonic chain with neutron number N=94.

With the resolution of the long-standing Lu puzzle, the authors also introduce a new method of significance to probe odd-even staggering in charge-radii (OEST) as a function of proton number, across isotonic chains where data remains scarce due to experimental challenges that the EBIT spectroscopy, in tandem with calculations, appear to resolve.

The manuscript of particular high quality, and could be accepted for publication in its current form given the efforts of the authors to accommodate the suggestions and comments of the reviewer. In particular, it is appreciated that the manuscript is now more self-contained, that

there is a more thorough presentation of the spectral data, and the risk of a logical fallacy is well explained and rebutted in the revised manuscript.

We thank the referee for the positive assessment of our work and for the helpful comments that improved the clarity and presentation of the manuscript.

Reviewer #3:

This work represents an outstanding achievement at the intersection of atomic and nuclear physics experiment. The authors have innovatively developed extreme ultraviolet (EUV) spectroscopy of highly charged ions (such as Na-like and Mg-like ions) using the Tokyo Electron Beam Ion Trap into a high-precision, cross-element tool for comparing nuclear charge radii. The experimental design is ingenious: Lu and Yb atoms of natural isotopic abundance are alternately injected via Knudsen cells, ionized to high charge states under identical EBIT conditions, and their D1 lines are recorded with a flat-field grazing-incidence spectrometer at a resolution of approximately 0.01 nm. To overcome spectrometer drift, the authors introduced calibration lines from W and Ne and established a time-dependent wavelength calibration model, thereby achieving stringent control over systematic errors.

The transition energy differences between Lu and Yb derived from the two independent spectral lines (Na-like and Mg-like) are compared with precise atomic structure calculations based on relativistic many-body perturbation theory combined with QED corrections. By solving the scaled radius difference equation, the authors determined the charge radius of ^{175}Lu to be 5.291(11) fm, representing a downward revision of approximately 0.08 fm from the previous recommended value. This result definitively proves that the nuclear charge radius of ^{175}Lu is smaller than that of ^{174}Yb, thereby resolving the decades-old "Lu inversion anomaly" and restoring the normal odd-even staggering pattern—where odd-proton nuclei are more compact—in the N=94 isotonic chain. This study provides a novel and precise probe for nuclear structure research. While it should be acknowledged that the authors and their research team have previously demonstrated aspects of this methodology in earlier publications, this does not diminish the significance of its application in the present work. On the contrary, the maturity and reliability of the technique, as evidenced by its prior development, lend greater confidence to the exceptional precision and robustness of the current cross-element measurement. The novelty here lies not in the first demonstration of the tool, but in its deployment to decisively solve a longstanding nuclear structure puzzle and to reveal a striking new anomaly, thereby fully realizing its potential as a transformative probe in nuclear physics.

Another highlight of the paper is the claim of discovering an "unexpectedly large" enhancement of odd-even staggering specific to the N=94 isotonic chain and the assertion that mainstream Density Functional Theory (DFT) "fails" in this region. Currently, this remains

primarily a description of the phenomenon rather than a physical explanation. This finding is clearly worthy of further investigation.

In summary, this is a very solid piece of work, and the supplementary material provided by the authors is quite detailed. The authors have also addressed the previous reviewers' concerns with appropriate revisions and responses. That said, I acknowledge that the study's impact and depth may be viewed differently by some readers. Nevertheless, in my view, the manuscript is suitable for publication in NC.

We thank the referee for the positive and thoughtful assessment of our work.

Reviewer #4

I would like to focus on the technical aspects of the manuscript. Despite the modifications made by Authors, the resubmitted manuscript still contains several noticeable issues:

We address the referee's technical comments point by point below.

1) There is a well-established terminology in the field, according to which the Authors have measured a relative radius. As can be seen from the reports of other referees, the terminology used in the manuscript is indeed confusing. I therefore recommend to revise it accordingly, and the results are not referred as "absolute" radii, but consistently as relative ones. E.g. Page 2, l.c:

"When anchored to muonic atom data for Yb [31], our result provides a new value of the absolute nuclear charge-radii of Lu isotopes, including 175Lu" - here, absolute should be replaced by relative.

(The same part in the Authors' response reads as: "By combining our result with muonic-atom data, we infer a new value of the absolute nuclear charge-radii of Lu isotope..." - same problem).

Our primary experimental result is a relative (scaled) charge-radius difference. When anchored to external data, this result yields an absolute charge radius for Lu. We have clarified this distinction in the manuscript and note that the inferred absolute values may inherit the limitations of the underlying reference, as discussed in our response to Reviewer #1.

"These absolute values may inherit the limitations of the underlying reference, in this case those associated with charge-radius extraction from muonic-atom measurements in deformed nuclei."

For clarity, we have also explicitly referred to the scaled radius difference as a relative observable in the manuscript:

"This equation shows that the key observable is not the simple radius difference $R_B - R_A$, but rather a scaled (relative) radius difference, $D_{BA} \equiv R_B - (S_A/S_B)R_A$."

2) It is still strongly recommended that uncertainties associated with the reference radius be explicitly included in the uncertainty budget. For example, the reported value R[175Lu]=5.291(11)fm should be written as R[175Lu]=5.291(6)(10)fm, where the first set of parentheses reflects the uncertainty of R[174Yb]=5.3108(60) fm taken from Angeli (2013), and the second one correspond to the current work (in this case, the total uncertainty would effectively be 12 instead of 11, possibly a rounding issue).

We have adopted the referee's suggestion and now explicitly separate the uncertainty associated with the reference radius from that of the present work:

"This yields a ^{175}Lu radius of 5.291(6)(10)~fm, where the first uncertainty reflects the reference radius and the second the present work, and an isotonic difference of R_{175Lu}-R_{174Yb}= -0.017(10) fm."

2a) the reported value R[175Lu]=5.291(11)fm appears to be in disagreement with the value of 5.3700(304) reported in Angeli (2013). Any comments?

The apparent discrepancy reflects the combination of data used in the Angeli (2013) evaluation, which relies on muonic-atom measurements for Yb and electron-scattering results for Lu. As discussed in Sec. S1 of the Supplementary Information, this combination leads to the inverted odd-even staggering. Our result provides a direct constraint on the Lu-Yb radius difference and resolves this inconsistency, yielding a smaller Lu radius.

3) The added table S1 is very useful. It provides a much clearer understanding of which contributions are included in the calculations and which are not, and also raises some questions. Commonly, when extracting rms radii from isotope shifts, additional contributions should be analyzed, namely: higher-order term proportional to <r^2>^2, nuclear deformation <r^4>, higher-order nuclear recoil and nuclear polarizability. H.O. nuclear recoil is negligible here, as obvious from the first-order recoil contribution in the table. H.O. <r^2>^2 term is in some sense corresponds to my previously raised concern about the linearity across the considered range 94-104, and now somehow discussed. So,

3a) It would be desirable to evaluate this contribution explicitly, following the standard procedures used in isotope-shift analyses.

The second-order size correction was already included in the original manuscript. We have now expanded and clarified this discussion in Sec. S4 B of the Supplementary Information. Following Eq. (S4), we write $\delta E = S\delta R + S_2(\delta R)^2$, where S_2 is determined from a finite-field fit around the expansion point. We find that the resulting second-order contribution leads to

a negligible correction to both the transition energies and the extracted radius, well below the overall uncertainty.

"We also consider the second-order size correction to Eq. (S4), by writing $\delta E = S\delta R + S_2(\delta R)^2$ (for $\delta t = \delta\beta_2 = 0$). From the five-point polynomial fit at BC–DF level, we obtain second-order sensitivity coefficients S_2 = −0.015 eVfm^{-2} for Yb and S_2 = −0.016 eVfm^{-2} for Lu. The final inferred shift in the Lu radius is approximately $\delta R = R - R_0 \approx 0.08$ fm (see Sec. S4 D), implying a second-order energy correction of $S_2(\delta R)^2 \approx 0.1$ meV, which is negligible for the present purposes. Another way of looking at this is to note that the error in our extraction of the scaled-radius correction for Lu, due to our neglecting the second-order shift, is $\delta R_2 \approx S_2(\delta R)^2/S \approx 0.0004$ fm, or $(\delta R_2)^2 \approx 0.2\ 10^{-6}\ \mathrm{fm}^2$. One sees from Table S5 that this radius error represents a negligible fraction of the total uncertainty budget in the scaled-radius difference. Moreover, this error in the radius determination can always be reduced simply by choosing the expansion point R_0 closer to the final inferred radius R. However, it is already negligible for the choice of expansion point made here.

The second way in which the neglect of second-order shifts can affect our analysis is in the natural-abundance averaging of Eq. (S6), which is based on the linear assumption. The most critical case here is the large range of stable Yb isotopes between 168Yb and 176Yb, for which $\delta R \approx 0.05$ fm [S4], corresponding to a second-order energy shift $S_2(\delta R)^2 \approx 0.04$ meV. This is again completely negligible for the present purposes. We conclude that the linear approximation in Eq. (S4) introduces negligible error."

3b) Nuclear deformation is another important contribution. The Authors starts with a deformed Fermi distribution. How do they treat the deformation among the isotope chain? Is the same \beta_2 used through it, or for every isotope the value of deformation is different? In any of those cases, is the approach fully consistent and free from double-counting?

The extraction of the scaled-radius difference, which constitutes the primary result, requires only the natural-abundance-averaged deformation parameter for each element. This quantity is used as the expansion point in Eq. (S4), together with the corresponding skin-thickness parameter, and their uncertainties are propagated as described in Sec. S4 A.

The discussion of individual isotopes is a separate post-processing step. In this stage, we use the generalized least-squares procedure described in Sec. S4 C, which combines the abundance-averaged result with optical isotope-shift data. No explicit assumption about isotope-dependent deformation is required.

3c) finally, nuclear polarizability is currently fully omitted. A justification is required, especially since unlike all other QED effects, NP is highly nucleus-specific and can not be expected to fully cancel in the considered difference. Moreover, due to the large uncertainty of the effect

itself, the remaining NP uncertainty in the isotonic difference can be orders of magnitude larger than the contribution itself. At minimum, estimates, clarifications, or justification should be provided.

We have expanded the discussion of nuclear-polarization effects in Sec. S3 B of the Supplementary Information. While we do not explicitly calculate this contribution, existing studies for highly charged ions indicate that nuclear polarization is comparable in magnitude to the nuclear-deformation effect and exhibits similar scaling with Z. In our analysis, deformation contributes at the sub-meV level to the transition-energy differences and has a negligible impact on the extracted scaled-radius difference and its uncertainty (see Secs. S4 B and S4 D, and Table S5). On this basis, we conclude that nuclear-polarization effects are likewise negligible for the present work.

"We have not explicitly estimated the nuclear-polarization contribution, which would require nuclear-structure calculations with considerable uncertainties. Recent work on nuclear polarization in the hydrogen-like $2s_{1/2}$ state of ^{208}Pb found a correction below 10 meV [S28], and an order of magnitude smaller for the $3s_{1/2}$ state. These results were subsequently used to estimate nuclear polarization in Li-like ions [S29], where it was shown to be comparable in magnitude to the nuclear-deformation effect, which exhibits a similar Z scaling. In the Na-like and Mg-like systems considered here, both nuclear-polarization and deformation effects are further suppressed. We find that the total nuclear-deformation contribution for the transitions studied here is at most 0.4 meV. We therefore consider both effects to be negligible at the present level of accuracy, as reflected in the uncertainties in Table S5. This result highlights an important distinction between our EUV method and the muonic-atom approach, for which both nuclear deformation and nuclear polarization produce large uncertainties in this region of the nuclear chart."

4) A minor comment: Sup. Mat., page 9: "Regarding the hyperfine effect, if the hyperfine levels in the EBIT are populated statistically, any hyperfine shift is expected to average to zero at the line center." This hold only at first order, what about second-order hfs? How large is it compared to the claimed measurements accuracy?

We estimated the dominant contributions to the second-order hyperfine shift within the 3s-3p multiplet (that is, arising from off-diagonal hyperfine matrix elements between the 3s, $3p_{1/2}$, and $3p_{3/2}$ states), finding it to contribute a very small shift to the D1 line centroid of about 0.01 meV:

"Regarding the hyperfine effect, if the hyperfine levels in the EBIT are populated statistically, any hyperfine shift is expected to average to zero at the line center. In contrast, a non-statistical population can introduce asymmetries in the line shape and cause a small shift in the measured energy, as can second-order hyperfine shifts. We estimate both effects to be below 0.1 meV in the transition-energy difference, rendering them entirely negligible."

5) The cancellation of uncertainties relies entirely on the assumption expressed in Eq. (S1), which itself is based on calculated-energies pattern. If certain contributions are missing, underestimated, or inaccurately computed, this approach would not reveal such deficiencies. I find the assumption (S1) over optimistic, and likely invalid over the considered range of Z=69-84. Even the energies themselves may exhibit a non-smooth behavior (e.g. due to NP contribution), and it is even more likely for the uncertainties (e.g. because of the different nuclear deformation parameter \beta_2 and its' uncertainty). I encourage the Authors to provide a convincing justification.

The referee's concern appears to stem from the role of Eq. (S1). This relation is not used to assign uncertainties, but rather to provide a simple estimate of their expected suppression. The actual uncertainty analysis is described in Sec. S3 B, where the uncertainties are assigned based on the behaviour of the calculated electronic contributions along the isoelectronic sequence.

As discussed there, the electronic contributions are smooth functions of Z, so any residual theoretical error can be represented as a smooth function ϵ(Z), and the uncertainty in a transition-energy difference is given by ϵ(Z)− ϵ(Z'). This procedure is validated by the observed smoothness of the calculated quantities (Fig. S4) and by the consistency between different evaluation methods described in Sec. S3 B.

Residual nuclear contributions (recoil, polarization, and hyperfine effects) are treated separately and are found to be negligible at the level of the present uncertainty budget. To clarify these points, we have expanded the discussion in Sec. S3 B and revised the introduction to Sec. S3 C in the Supplementary Information.

"Noting that the estimated uncertainties $\Delta E_f(Z)$ reliably track the true error across the entire isoelectronic sequence, we therefore assume that $|\epsilon(Z)|$ can be approximated by $\Delta E_f(Z)$ and assign an uncertainty of $|\Delta E_f(Z) - \Delta E_f(Z')|$ to the calculated transition-energy difference. We can also obtain equivalent results, to within an accuracy of better than 0.1 meV, by making a simple two-point subtraction of the estimated uncertainties, $|\Delta E(Z) - \Delta E(Z')|$, without first finding the fitted functions $\Delta E_f(Z)$. Likewise, the computed transition-energy differences can be obtained equivalently from either $E_f(Z) - E_f(Z')$ or $E(Z) - E(Z')$, to better than 0.1 meV. This happens because nuclear effects are small and our calculated electronic quantities are sufficiently free from potentially large random numerical effects."

First sentence of Sec. S3 C:

"In this section, we describe a simple way of understanding why in this case the absolute uncertainty in the transition-energy difference is suppressed relative to that in the transition energy itself."

In light of the points raised above, I regret to conclude that the presented data currently do not seems to support the conclusions and claims, especially when it comes to the

uncertainties. Several relevant contributions are either missing or insufficiently addressed in the theoretical analysis. The uncertainties budget is based on a seemingly empirical formula, which is not confirmed by a rigorous approach, likely leading to a significant underestimation of uncertainties. Therefore, the manuscript can not be published in its' present form.

Response to Reviewers

In what follows, we reproduce the reviewers' comments in blue and indicate our revisions in red. We also attach a PDF of the revised manuscript and SI with all changes marked in red.

Reviewer #1 :

In the revised version, my comments have been properly addressed, and I therefore recommend its publication now.

We thank the reviewer for their careful review of the manuscript, which helped us to improve the clarity of our presentation.

Reviewer #4 :

The Authors have made an effort to address my previous comments. While most points have been answered, some responses remain somewhat superficial. Therefore, I must request further clarification and rigor regarding the following points before this manuscript can be considered for publication:

1) First and foremost, all discussed contributions must be explicitly included in Table S1, including nuclear polarizability and nuclear deformation, along with both their estimated values and associated uncertainties. Omitting these data from the table gives the incorrect impression that they have been ignored in the analysis.

As requested, we have now included estimates of the nuclear-polarization effect in Table S1. Nuclear deformation has always been included implicitly in Table S1 and does not need a separate line, because we perform all calculations using a deformed nucleus (see table footnotes *a* and *b*).

2) "We have not explicitly estimated the nuclear-polarization contribution, which would require nuclear-structure calculations with considerable uncertainties. Recent work on nuclear polarization in the hydrogen-like 2s1/2 state of 208Pb found a correction below 10 meV [S28], and an order of magnitude smaller for the 3s1/2 state."
While I agree that an accurate calculation of NP is truly complex, a quantitative estimation must be provided. The reference to H-like systems is not entirely relevant here: because NP is a short-range interaction, core-excitation effects are highly significant. This means that the effect for an H-like 2s state can easily be an order of magnitude smaller than the NP effect for a Li-like system with an electron in the 2s state for the same nucleus. This effect is even more pronounced for 3s states. Additionally, it appears that the Authors have performed some preliminary estimations that are not shown in the manuscript. These estimations should be explicitly included.

The quoted section has been removed and is superseded by the discussion in point 4 below.

3) "These results were subsequently used to estimate nuclear polarization in Li-like ions [S29], where it was shown to be comparable in magnitude to the nuclear-deformation effect, which exhibits a similar Z scaling."
This statement is too vague, and more detailed information is required. What is the specific scaling being referenced? What are the calculated contributions for the reference systems? Most importantly, how do these translate into specific values for the HCIs measured in this work?

The quoted section has been removed and is superseded by the discussion in point 4 below.

4) "In the Na-like and Mg-like systems considered here, both nuclear-polarization and deformation effects are further suppressed."
This suppression holds true only if core-excitation effects are not-existing, which cannot be simply taken for granted.

We thank the reviewer for their interest in nuclear polarization (NP) and agree that the NP shift in highly charged ions (HCIs) is a challenging subject generally. In the original submission, we argued that the NP contribution was at the sub-meV level and not significant for this application. We expanded that discussion in the second revision and now, in this third revision, we include explicit estimates as requested. Our conclusion remains essentially unchanged: NP is expected to contribute at most several percent to the total uncertainty budget and is therefore not significant at the level relevant to the present analysis.

We originally excluded the small NP effect from Table S1 because the corresponding theory seemed to us incomplete. The discussion of NP in Sec. S3 B has now been revised and we also discuss briefly some of the outstanding theoretical issues (see below). The leading core-excitation effects are included by incorporating an effective polarization potential into the self-consistent Hartree-Fock (BC-DF) procedure; higher-order core-excitation effects are strongly suppressed in these HCIs.

"Among the other nuclear effects, the most uncertain contribution is the scalar nuclear-polarization (NP) shift. As an example of an explicit estimate of this shift, we apply the Coulomb-approximation method of Refs. S28 and S29, with the effective polarization potential of Ref. S29 included in the BC-DF procedure. We take the A-dependent fit to the nuclear electric-dipole (E1) polarizability $\alpha^{\mathrm{E1}}(A)$ from Ref. S30, and assume the approximate formula $B(E2) = (3/4\pi)^2 Z^2 e^2 c_{\mathrm{nuc}}^4 \beta_2^2$ for the reduced electric-quadrupole (E2) transition probability to the first-excited collective rotational state [S31], where c_{nuc} is the half-density nuclear radius and β_2 is the quadrupole deformation. For the Na-like D_1 transition in $^{174}\mathrm{Yb}$, using the nuclear data in Table S4, this procedure gives an NP-E1 shift of 0.23 meV and an NP-E2 shift of 0.33 meV, for a total NP shift of 0.56 meV.

In Sec. S4, we use the natural-abundance-averaged Lu-Yb transition-energy difference to extract the scaled-radius difference. In this Lu-Yb difference, the NP contribution is expected to cancel systematically to some extent. If we apply the method described above to the stable

Lu and Yb isotopes, we obtain an NP difference of $\delta(\mathrm{NP}) \approx 0.04$ meV. This procedure likely overestimates the NP cancellation, however, because the relevant nuclear parameters may vary somewhat irregularly between neighboring nuclei, rather than follow the smooth parametrizations assumed here. Also, the formula for $B(E2)$ strictly applies only to $0^+ \rightarrow 2^+$ transitions in even-even nuclei. We therefore assign a theoretical uncertainty to $\delta(\mathrm{NP})$ that is a significant fraction of the NP shift itself. In addition, Refs. S32 and S33, which use nuclear density-functional theory (DFT), suggest that including excitations to additional nuclear states, together with transverse-photon terms, can increase the NP shift by roughly a factor of two in this range of Z. Such DFT calculations have not yet been performed for the highly deformed Lu or Yb isotopes. Nevertheless, even after accounting for these considerations, it is reasonable to conclude that the theoretical uncertainty in $\delta(\mathrm{NP})$ enters at the sub-meV level. In Table S1, we assign an uncertainty of 1 meV to the NP shift in a transition energy, and an uncertainty of 0.5 meV to $\delta(\mathrm{NP})$.

The analysis in Sec. S4 B and Table S5 shows that a 0.5 meV uncertainty in $\delta(\mathrm{NP})$ corresponds to only 3% of the total uncertainty budget. We therefore conclude that NP shifts are not significant for the present application, even if it turns out that we have slightly underestimated the true errors. This result highlights an important difference between the present EUV method and the muonic-atom approach, for which nuclear polarization (and deformation) can introduce significant uncertainties in this region of the nuclear chart."

5) "We find that the total nuclear-deformation contribution for the transitions studied here is at most 0.4 meV. "
Please justify the origin of this value. As currently written, this upper bound appears unsupported by data or citations.

Taking the 10 meV value cited by the Authors, scaling it up for core-excitation effects, and then down for the 3s state, it is entirely plausible that the final value of the NP contribution remains on the order of a few meV. According to the Authors', a similar value should be assigned to nuclear ND. Based on the justifications presented in the manuscript, I am not yet convinced that these contributions are truly negligible.

The quoted section has been removed and is superseded by the discussion in point 4 above. We have always included nuclear deformation nonperturbatively throughout the RMBPT and QED calculations. This was the basis for our earlier estimate of 0.4 meV for the overall deformation shift, which includes core excitation to high order.

6) The correctness of the results is still being argued based on the assumption that the neglected nuclear effects are small. However, even if these neglected effects were significant, omitting them would likely still produce a smooth curve. Therefore, the smoothness does not serve as a valid justification for neglecting them.

Note that the quantity $E_{\Delta}(Z)$ defined in Sec. S3 B involves subtracting the BC-DF energy, E_{DF}. Since E_{DF} contains the leading NP and recoil contributions, as well as the leading

nuclear-size correction, the correction $E_\Delta(Z)$ is indeed very smooth and the argument in that section is therefore essentially correct.

To make this scrupulously clear, we have made a number of small modifications to that discussion (see attached file of SI with modifications in red). The principal ones are:

"In Fig S4, we show $E_\Delta(Z) = E(Z) - E_{\mathrm{DF}}(Z)$, the difference between the calculated D_1 transition energies $E(Z)$ and the corresponding Dirac-Fock baseline value $E_{\mathrm{DF}}(Z)$, which already incorporates the leading nuclear-size, recoil, and NP corrections. Irregularities in $E_\Delta(Z)$ as a function of Z may arise from residual nuclear-size effects, other residual nuclear contributions, or numerical uncertainties in the calculations. Thus, the transition-energy correction $E_\Delta(Z)$ is principally an electronic quantity and largely free of irregularities associated with nuclear effects."

and, a little lower down, we have inserted:

"Using this subtractive procedure, we can assign uncertainties to all electronic-structure quantities in Table S1. The nuclear recoil, polarization, and hyperfine effects are assigned uncertainties separately, and all uncertainties are then added in quadrature."

All of these issues could likely be resolved by adopting a more conservative approach to the error estimations. Incorporating these uncertainties would preserve the impact of the work while ensuring it remains scientifically rigorous.

NP uncertainty from Table S1 has now been fully included in the analysis, leading to a ~1.5% increase in the standard deviation of the scaled-radius difference. The change in the radius uncertainties, at the quoted level of precision, is 0.013 fm --> 0.014 fm for 175Lu, which is mainly a rounding-error effect.

Based on the points raised above, I stay of the opinion that the underlying theory requires further refinement to robustly support the current claims. Consequently, I cannot recommend the manuscript for publication in its current form without these necessary improvements.

Response to Reviewer #4 and the Editor

We thank the reviewer for the careful evaluation of our revised manuscript and for recommending publication.

The remaining revisions in this submission address the editorial and production requests outlined in the editor's checklist. No further scientific changes to the manuscript were requested by the reviewer.

We thank the editor and reviewer for their time and consideration.
